# Health of Black Populations and Sexual and Gender Minorities in Health Education: A Scoping Review

**DOI:** 10.3390/nursrep16070231

**Published:** 2026-07-02

**Authors:** Bruno Pereira da Silva, Patrícia de Carvalho Nagliate, Gabriel da Silva Brito, Danilo Bonfim Sousa de Queiroz, Ana Paula de Morais e Oliveira, Célia Alves Rozendo, Danielly Santos dos Anjos Cardoso, Giovanne Bento Paulino, Ygor de Oliveira Navarro da Conceição, Renata Soares da Luz, Fernanda Mota Rocha, Dalvani Marques, Danielle Satie Kassada, Roberto Ariel Abeldaño Zuñiga, Paula Cristina Pereira da Costa, Maria Giovana Borges Saidel, Eduardo Sodré de Souza, Débora de Souza Santos

**Affiliations:** 1 Multidisciplinary Center, Federal University of Acre (UFAC), Cruzeiro do Sul 69890-000, AC, Brazil; 2 School of Nursing, State University of Campinas (UNICAMP), Campinas 13083-887, SP, Brazil; g263594@dac.unicamp.br (G.B.P.); y272647@dac.unicamp.br (Y.d.O.N.d.C.); r160240@dac.unicamp.br (R.S.d.L.); fmrocha01@gmail.com (F.M.R.); dalvani@unicamp.br (D.M.); dkassada@unicamp.br (D.S.K.); paulapc@unicamp.br (P.C.P.d.C.); mgsaidel@unicamp.br (M.G.B.S.); edusodre@unicamp.br (E.S.d.S.); 3 School of Philosophy, Languages and Literature, and Human Sciences, Federal University of São Paulo (UNIFESP), Guarulhos 02141-000, SP, Brazil; danilo.bonfim@unifesp.br; 4 School of Nursing, Federal University of Alagoas (UFAL), Maceió 57000-000, AL, Brazil; patricia.nagliate@eenf.ufal.br (P.d.C.N.); celia.rozendo@gmail.com (C.A.R.); danielly.anjos@eenf.ufal.br (D.S.d.A.C.); 5 Faculty of Education, University of São Paulo (FEUSP), São Paulo 05508-040, SP, Brazil; g.brito@usp.br; 6 Library of the Faculty of Medical Sciences, State University of Campinas (UNICAMP), Campinas 13000-000, SP, Brazil; arvigui@unicamp.br; 7 Department of Nursing Science, Faculty of Health Sciences, University of Eastern Finland, FI-70210 Kuopio, Finland; ariabeldanho@gmail.com

**Keywords:** continuing education, professional training, teaching, Black people, sexual and gender minorities, health inequalities, scoping review

## Abstract

**Objective**: To map the scientific evidence and identify knowledge gaps regarding the health of Black and Sexual and Gender Minority populations within the global context of health education. **Introduction**: Health education curricula should explicitly recognize, define, and address the specific needs and health disparities affecting Black and Sexual and Gender Minority populations to ensure that healthcare provision is comprehensive and inclusive in diverse settings. **Eligibility criteria**: Studies related to professional health training at undergraduate and graduate levels, as well as other educational modalities addressing healthcare provision for Black and Sexual and Gender Minority populations, were included. **Methods**: This scoping review was conducted following the JBI methodology. Studies were retrieved from Scopus, Web of Science, PubMed, Embase, MEDLINE, Virtual Health Library, CINAHL, ERIC, Cochrane Library, Brazilian Digital Library of Theses and Dissertations, ProQuest Dissertations & Theses Global, EBSCO databases, and the Networked Digital Library of Theses and Dissertations, without language or time restrictions. Two independent reviewers screened the studies and extracted data using a standardized form developed for this review. Concepts, definitions, structures, results, and applications of professional health education for the care of Black and Sexual and Gender Minority populations were systematically synthesized. The results were organized and presented in tabular and graphical formats, accompanied by a narrative summary. **Results**: A total of 104 studies were included. The evidence was predominantly concentrated in North America, particularly in the United States, with limited representation from other regions. Most studies were published after 2020, indicating a recent expansion of research interest. The methodological profile was characterized by a predominance of quantitative and descriptive designs, alongside qualitative and mixed-methods approaches. Thematic analysis revealed a concentration of studies addressing gender-affirming care, workforce diversity, social determinants of health, and discrimination, while intersectional approaches and long-term educational outcomes remained less explored. **Conclusions**: The available evidence indicates that health education has increasingly incorporated themes related to equity and diversity; however, the integration of structured and mandatory curricular approaches addressing the intersectional health needs of Black and Sexual and Gender Minority populations remains limited. The findings highlight the need for broader geographic representation, stronger methodological designs, and the development of comprehensive educational strategies capable of addressing structural inequalities within health training contexts.

## 1. Introduction

The vulnerability experienced by Black populations and Sexual and Gender Minorities (SGM) positions these groups among those with the most unfavorable health outcomes. This condition is not incidental but rooted in structural inequalities that constrain access to resources and limit the availability of protective strategies, reflecting the broader social determination of the health–disease process [1]. Within this framework, health education emerges as both a site of reproduction and a potential locus of transformation of such inequities, as its gaps frequently mirror the structural exclusions that shape health outcomes.

An intersectional perspective provides a critical lens through which these dynamics can be examined, particularly in relation to the marginalization of race, gender, and sexuality within health curricula. The relative invisibility of these dimensions contributes to the persistence of preventable illness and premature mortality among Black and SGM populations. Expanding beyond a strictly biomedical orientation, intersectionality enables the incorporation of structural determinants that configure unequal health experiences.

The choice to focus on Black and SGM populations is not arbitrary; it stems from the recognition that these groups occupy central axes of exclusion in Western medical history—namely, the colonial/racial hierarchy and the cis-heteronormative binary. By analyzing these two groups, this review addresses the structural roots of what the World Health Organization identifies as key social determinants of health that sustain global health inequities.

In line with terminology commonly used by the National Institutes of Health (NIH), this review adopts the term Sexual and Gender Minorities (SGM) to refer to individuals whose sexual orientation, gender identity, gender expression, sex characteristics, or sex development differ from dominant cis-heteronormative norms. The term is used throughout this review as the primary analytical category.

An intersectional and Critical Social Theory perspective is essential to understanding how race, gender, and sexuality shape health inequalities. Structural racism and cis-heteronormativity contribute to persistent disparities, which remain insufficiently addressed in health education curricula [2,3,4,5,6].

Similarly, SGM populations face barriers rooted in institutionalized discrimination, often conceptualized as SGM-related hostility, which directly affects access to care, health promotion, and disease prevention [6]. These structural constraints underscore the importance of health training capable of responding to inequities produced by racism, cis-heteronormativity, socioeconomic adversity, and institutional discrimination. Although such an approach has been emphasized in the literature [1], it remains insufficiently incorporated into undergraduate curricula, limiting the capacity of health professionals to address complex and intersectional needs [7,8]. When present, content related to Black and SGM health is frequently treated as peripheral, lacking systematic or mandatory integration [9,10], which compromises the provision of comprehensive and humanized care [8,11].

A monolithic approach to healthcare professional training contributes directly to the persistence of practices that fail to achieve comprehensive care and to respond to the specific needs of each group, especially when considering the compounding systems of oppression such as race, gender, and class. In this regard, it is worth noting that medical and health education must urgently overcome the reproduction of these inequities. Such specificities call for deeply intersectional analyses and critical educational concepts and practices.

International evidence further substantiates the persistence of health inequalities affecting these populations. Structural racism and discrimination based on sexual orientation and gender identity are recognized as key social determinants of health, contributing to barriers in access to services, higher disease burden, and reduced life expectancy [12,13]. These patterns reinforce the need for intersectional approaches in health education that simultaneously address ethnic-racial and gender-based dimensions, in alignment with global commitments to reducing health inequities.

In this context, health practice requires a political and ethical commitment to valuing life, integrating technical competence with cultural sensitivity. Health education should integrate technical competence with culturally responsive, socially accountable, and equity-oriented approaches [4,14,15,16]. However, the limited integration of these approaches into existing curricular frameworks undermines the development of professional competencies necessary to address the demands of marginalized populations [17]. The absence of intersectional content in training exacerbates health disparities, reinforcing the effects of overlapping systems of oppression, including racism, sexism, and classism [6,10,16]. Addressing these gaps requires the intentional integration of these themes into undergraduate, postgraduate, and continuing education programs.

Preliminary searches in major databases did not identify ongoing or completed reviews on this topic. Existing studies in Brazil highlight significant gaps in the inclusion of Black and SGM health in professional training, whether in medical curricula [18], public health [17], or institutional policies [19]. These findings underscore the need for broader investigations that consider diverse geographical and cultural contexts.

In contrast to these studies, the present scoping review proposes an international review focused on the training of current and future health professionals. By mapping the available evidence and identifying knowledge gaps, this review seeks to advance the understanding of how structural inequalities, shaped by intersectional dynamics, influence health education globally [2,3].

Crucially, while this review maps evidence related to Black and SGM health, it does not treat these as monolithic or mutually exclusive categories. On the contrary, adopting an intersectional framework allows us to critically examine how the literature itself often fails to account for the unique health experiences at the intersections of race, gender, and sexuality (e.g., Black transgender individuals or Black queer women). Thus, this scoping review seeks to identify whether health education is moving toward an integrative understanding of these overlapping systems of oppression or if it remains limited to additive and fragmented pedagogical approaches.

The aim of this scoping review is to map scientific evidence and identify knowledge gaps regarding how the health of Black and/or SGM populations has been addressed in the training of health professionals worldwide, with particular attention to how intersectional dynamics are incorporated or silenced in these educational processes.

### Review Questions

Based on the objective of this review, two guiding questions emerge for the scoping review:

1. What is the current scientific evidence regarding the health of Black and/or SGM populations in the training of current and future healthcare professionals worldwide?
2. What are the existing knowledge gaps in this area?

## 2. Inclusion Criteria

### 2.1. Population

This scoping review encompassed empirical studies evaluating health professionals and prospective health professionals exposed to educational interventions addressing healthcare for Black and/or SGM populations across undergraduate, graduate, and continuing education levels. Eligible educational modalities included technical and vocational training, permanent education, short-term or certification courses, on-the-job learning, and institutional initiatives that explicitly integrated these population groups into health curricula or professional development frameworks [14]. Institutions and programs that implemented relevant health education strategies were also considered eligible.

Health education was defined as the systematic process of developing the knowledge, competencies, and values required for the professional practice of health workers and trainees. In this review, health education included undergraduate training, stricto and lato sensu graduate education, continuing education, permanent education, technical and vocational training, certification courses, and workplace-based learning, particularly in international contexts characterized by diverse professional development infrastructures. The educational process under review encompassed pedagogical approaches, curricular design, teaching methodologies, and policy frameworks aimed at aligning professional training with the evolving demands of health systems and society. This process is anchored in experiential learning, knowledge generation, and creative practice, fostering comprehensive, humanized, and high-quality care [14,20,21].

Within the analytical framework guiding this review, the quadrilateral of health education comprises four interconnected domains: teaching, management, care, and social control. Teaching refers to pedagogical and curricular processes; management to health service organization and policy development; care to comprehensive and equitable healthcare delivery; and social control to societal engagement in health policy planning and oversight. This integrative model supports health professional training that is responsive to health system requirements and community needs [14].

Professional training in the health sector is not limited to occupational preparation; it also cultivates interpersonal skills, such as active listening, that underpin effective provider-user interactions and high-quality care. Permanent Education in Health (PEH) constitutes a strategic mechanism for ongoing professional development and systematic improvement of health workforce capabilities [14].

PEH is conceptualized as an educational process that fosters critical reflection on daily practices through the problematization of work processes. Its principal objective is to transform both professional practices and service organization, thereby responding to the needs of the population, health system management, and social control imperatives. PEH facilitates continuous professional development by incorporating advances in theory, methodology, science, and technology [22].

PEH is aligned with multiple educational paradigms: Education in Service involves institutional and policy-driven modifications in technical training; Continuing Education emphasizes context-specific professional advancement; and Formal Education integrates workplace experiences with curricular initiatives in health workforce development [22].

### 2.2. Concept

The principal concept guiding this review is the health of Black populations and Sexual and Gender Minorities (SGM).

Black populations are understood as racialized social groups whose identification is often associated with phenotypic characteristics, including skin tone, hair texture, and facial features. Racial classification is not a natural fact but a historically situated social construction, and the criteria used to define who is considered Black vary across national contexts. Therefore, any cross-context interpretation of this category must remain attentive to distinct classificatory systems and racial formations [23].

In this review, SGM is adopted as the primary terminology to encompass individuals whose sexual orientation, gender identity, gender expression, sex characteristics, or sex development differ from dominant cis-heteronormative norms. We acknowledge that the international literature employs a variety of overlapping terms, including LGBTQIA+ and LGBT+. For conceptual consistency, these terms were treated as related descriptors during the search process, while SGM was used as the overarching analytical category throughout the review [23,24].

The field of Black population health examines disparities arising from racism, socioeconomic adversity, and unequal access to healthcare, including elevated maternal and infant mortality, higher prevalence of chronic and infectious diseases, and the consequences of violence [4]. Correspondingly, the health needs of SGM communities remain underserved, largely due to institutionalized discrimination and broader structural factors, such as poverty and health inequities.

This field promotes strategies aimed at comprehensive healthcare, including targeted public policies, workforce qualification, continuing professional education, and expanded access to health services, particularly in response to exclusion and violence within health and social protection systems [20].

Black and SGM populations experience vulnerabilities operating at individual, community, and structural levels, often intensified by institutional neglect and systemic inequities. These persistent disparities, rooted in racism, sexism, cis-heteronormativity, and other intersecting systems of oppression, underscore the need for inclusive and transformative public policies capable of advancing equity, social justice, and the right to health [25].

### 2.3. Context

Adhering to JBI methodology, this review imposed no geographical restrictions; studies addressing health education in any country or region were eligible for inclusion [26].

Differences in health system organization influence the structure and implementation of health education across countries, reflecting distinct historical, political, economic, and social contexts. Health systems vary in their approaches to financing, governance, service delivery, and workforce development, which may shape educational priorities and opportunities related to Black and SGM health [27].

These structural differences are particularly relevant when considering the health needs of populations historically affected by social inequities. In universal health systems, comprehensive care is typically supported by coordinated service networks, strong primary healthcare systems, and public sector stewardship. In contrast, more market-oriented systems often rely on a greater degree of private financing and service provision, which may influence patterns of access, coverage, and health workforce training [27,28,29].

Given the international scope of this review, studies were included regardless of the health system model in which they were conducted, encompassing universal public systems, mixed public–private arrangements, social insurance models, and predominantly private healthcare systems [16].

The review is informed by principles of equity, universality, and social justice in health; however, its objective is not to compare or evaluate health system models, but rather to map how the health of Black populations and SGM communities has been addressed within health education across diverse contexts.

## 3. Types of Evidence and Study Designs

The review encompassed descriptive observational designs, including case series, individual case reports, and descriptive cross-sectional studies, as well as analytical observational studies, such as prospective and retrospective cohort studies, case–control investigations, and analytical cross-sectional analyses. Qualitative research methodologies were included, covering phenomenological inquiry, grounded theory, ethnography, qualitative description, action research, and feminist research. Systematic reviews relevant to the research question and meeting the eligibility criteria were considered. Theses, dissertations, and documentary surveys were also eligible for inclusion. Experimental or quasi-experimental studies were included when the intervention focused on health professional education or training related to Black and/or SGM populations. Clinical trials unrelated to educational processes, opinion articles, and non-research texts were excluded.

### 3.1. Methods

The scoping review was conducted in accordance with JBI methodological guidance for scoping reviews [26] and was reported following the PRISMA-ScR checklist [30]. The review was based on a previously published protocol [31].

Evidence syntheses are embedded within a broader evidence ecosystem, serving as essential components of care and evidence-based research [26,32]. JBI endorses eight distinct review methodologies, which collectively comprise this ecosystem.

Scoping reviews are designed to map foundational concepts, clarify definitions and conceptual boundaries, explore the breadth of the literature, and synthesize evidence to inform future research. No assessment of methodological limitations or risk of bias is performed, as the primary aim is to provide an overview of available evidence and identify gaps. Scoping reviews are often precursors to systematic reviews [26,32].

### 3.2. Deviations from Protocol

No deviations from the previously published protocol were identified during the conduct of this scoping review. All procedures were carried out in accordance with the methodological framework and eligibility criteria established in the protocol [31].

### 3.3. Search Strategy

The search strategy was designed to capture both published and unpublished literature (grey literature). A three-stage approach was implemented: (1) an initial search, guided by a professional librarian, in MEDLINE (Ovid), PubMed, Scopus (Elsevier), and CINAHL to identify relevant articles and extract key terms from titles, abstracts, and indexing; (2) the development and adaptation of comprehensive search strategies for MEDLINE (PubMed), Scopus (Elsevier), and CINAHL (EBSCOhost), as detailed in the published protocol [31]; and (3) screening of the reference lists of included studies to identify additional sources.

The search strategy included studies published in any language and without temporal restrictions. Keywords and controlled vocabulary were adapted for each database to optimize retrieval. Reference lists of included studies were also screened.

Information sources included Scopus (Elsevier), Web of Science (Clarivate), MEDLINE (Ovid), PubMed (National Library of Medicine—NLM), PubMed PMC (National Library of Medicine/National Institutes of Health—NIH/NLM), Embase (Elsevier), Virtual Health Library (VHL/Biblioteca Virtual em Saúde; BIREME/PAHO/WHO), CINAHL (EBSCOhost), ERIC (EBSCOhost), and the Cochrane Library (Wiley). Grey literature sources included the Brazilian Digital Library of Theses and Dissertations (BDTD; Instituto Brasileiro de Informação em Ciência e Tecnologia—IBICT), the Networked Digital Library of Theses and Dissertations (NDLTD), and ProQuest Dissertations & Theses Global (PQDT; Clarivate).

### 3.4. Study Selection

Upon completion of the search, all records were consolidated and imported into EndNote Web for duplicate removal [32]. Following pilot testing, two independent reviewers screened titles and abstracts against predefined eligibility criteria. Full texts of potentially relevant studies were retrieved and imported into Rayyan QCRI for detailed assessment [33].

**Figure 1:**
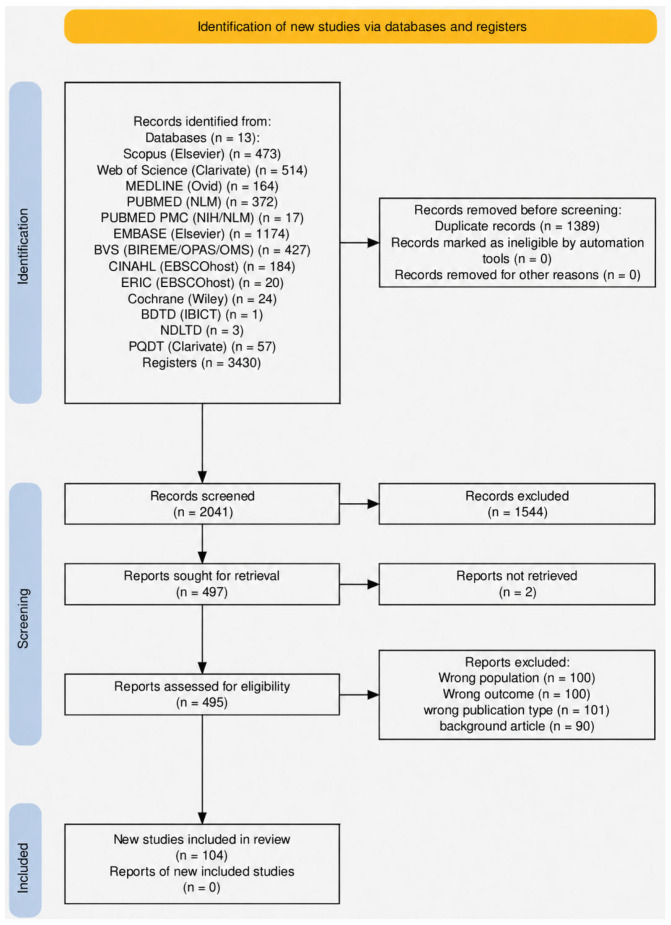
PRISMA flow diagram of the study selection process for the scoping review (n = 104). Created using the website https://estech.shinyapps.io/prisma_flowdiagram/ (accessed on 25 May 2026) [34]. Interpretation: Of the 3430 records identified, 104 studies met the eligibility criteria and were included in the scoping review. The most significant reduction occurred during the title and abstract screening, while mismatches in publication type, population, and outcome were the main reasons for exclusion at the full-text stage. BVS = Biblioteca Virtual em Saúde/VHL = Virtual Health Library; BDTD = Brazilian Digital Library of Theses and Dissertations; NDLTD = Networked Digital Library of Theses and Dissertations; PQDT = ProQuest Dissertations & Theses Global; NLM = National Library of Medicine; NIH/NLM = National Library of Medicine/National Institutes of Health.

Two reviewers (DBQ and GSB) independently assessed the full texts of selected studies for inclusion and documented the reasons for excluding ineligible studies. Discrepancies at any stage were resolved through discussion, with arbitration by a third, more experienced reviewer (PCN) when necessary. The study selection process, including all search results and inclusion decisions, is presented in Figure 1 using a PRISMA flow diagram [30,34].

## 4. Data Extraction

Data extraction was performed independently by two or more reviewers using a tailored extraction instrument developed by the review team. Extracted data encompassed participant characteristics, conceptual focus, contextual details, methodological features, and principal findings relevant to the review question.

Distinct extraction forms were created for each source type considered, as outlined in Supplementary Material SI. The extraction tool was iteratively refined as necessary throughout the process, and all modifications were systematically documented (Supplementary Matrial SII).

Reviewer disagreements were resolved by consensus or, when required, by consulting additional reviewers. Study authors were contacted when additional or supplementary information was required. The extraction forms were pilot-tested on a sample of studies to ensure comprehensive and accurate data capture.

To facilitate the identification of knowledge gaps, a thematic gap matrix was constructed, categorizing included studies by geographic region (North America, South America, Europe, Africa, Asia, Oceania, Multiregional/Global) and educational level (technical training, undergraduate, postgraduate, and continuing education). This matrix provides a visual representation of underrepresented areas in the literature and helps inform future research priorities (Supplementary Material SIII).

## 5. Data Analysis and Presentation

Extracted data were synthesized and presented in tabular and diagrammatic formats, consistent with the review objectives. A descriptive summary accompanied the tabular results, contextualizing the findings with respect to the review’s aim and research questions.

**Figure 2:**
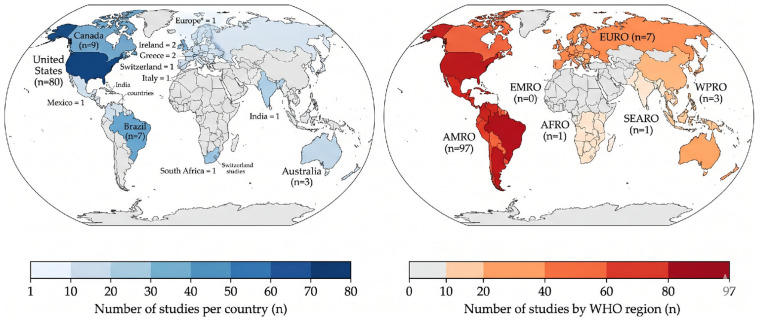
Geographic distribution of included studies. Global distribution of the studies included in the scoping review (n = 104), presented by country and aggregated according to World Health Organization (WHO) regions. **Abbreviations:** AFRO, African Region; AMRO, Region of the Americas; EMRO, Eastern Mediterranean Region; EURO, European Region; SEARO, South-East Asia Region; WPRO, Western Pacific Region. Europe* refers to the multicountry study by Chandel et al. [35], which reported Europe as a study location without specifying individual European countries; therefore, it was represented as an aggregated regional location rather than as a country-level count.

**Figure 3:**
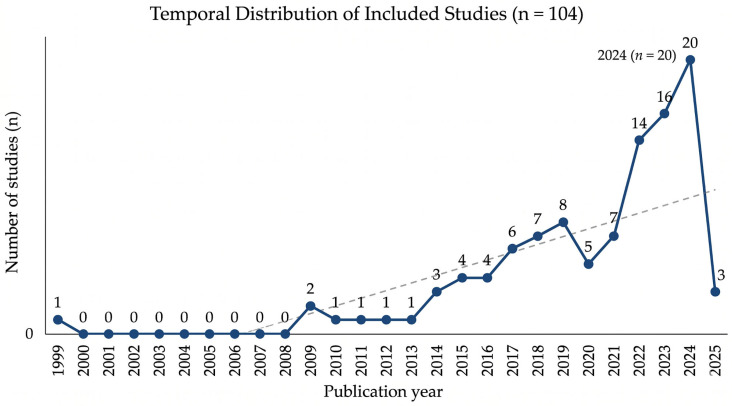
Temporal distribution of publications. Annual distribution of publications included in the scoping review (n = 104), covering the period from 1999 to 2025. The numerical labels above the markers indicate the number of studies published in each year, and the dashed gray line represents the linear trend fitted to the annual publication counts.

**Figure 4:**
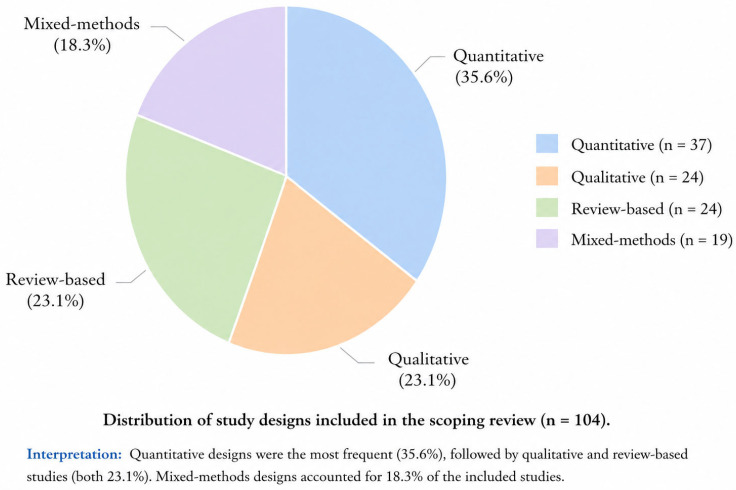
Methodological profile of included studies. Distribution of study designs among the studies included in the scoping review (n = 104), categorized as quantitative, qualitative, review-based, and mixed-methods. Percentages may not total 100.0% exactly due to rounding.

Figure 1 presents the PRISMA flowchart detailing the study selection process. Figure 2 illustrates the global distribution of the studies included in the scoping review, disaggregated by country and aggregated according to World Health Organization (WHO) regions. Figure 3 depicts the temporal distribution of publications spanning the period from 1999 to 2025. Figure 4 presents the methodological profile of the included studies, encompassing quantitative, qualitative, mixed-methods, review-based, and other study designs. Table 1 provides a thematic synthesis of the included studies, organized by thematic category, population, type of evidence, key findings, and corresponding studies.

## 6. Results

### 6.1. Study Selection

The PRISMA flow diagram, Figure 1, illustrates the study selection process of this scoping review, beginning with the identification of a total of 3430 records from 13 databases. After the removal of 1389 duplicate records, 2041 unique records were moved to the screening stage. Following initial screening and the subsequent assessment of 497 reports for eligibility, the process concluded with the final inclusion of 104 new studies in the scoping review.

### 6.2. Geographic Distribution of Studies

Most studies were conducted in the United States (n = 80) [35,36,37,38,39,40,41,42,43,44,45,46,47,48,49,50,51,52,53,54,55,56,57,58,59,60,61,62,63,64,65,66,67,68,69,70,71,72,73,74,75,76,77,78,79,80,81,82,83,84,85,86,87,88,89,90,91,92,93,94,95,96,97,98,99,100,101,102,103,104,105,106,107,108,109,110,111,112,113,114,115,116], representing the majority of the included literature (n = 104). A smaller number of studies were identified in Canada (n = 9) [35,36,117,118,119,120,121,122,123] and Brazil (n = 7) [124,125,126,127,128,129,130], followed by Australia (n = 3) [35,131,132]. Ireland and Greece each contributed two studies (n = 2) [133,134,135,136], while South Africa, Switzerland, Italy, India, and Mexico each accounted for one study (n = 1) [37,137,138,139,140]. Additionally, one study was classified as multicountry and reported Europe as its location (n = 1) [35]. In total, 12 country or regional classifications were identified across the included studies. The highest concentration of studies was observed in the United States, followed by Canada and Brazil, with all other countries contributing a limited number of publications.

The distribution by World Health Organization (WHO) regions shows a predominance of studies in the Region of the Americas (AMRO; n = 97) [35,36,37,39,41,42,43,44,45,46,47,48,49,50,51,52,53,54,55,56,57,58,59,60,61,62,63,64,65,66,67,68,69,70,71,72,73,74,75,76,77,78,79,80,81,82,83,84,85,86,87,88,89,90,91,92,93,94,95,96,97,98,99,100,101,102,103,104,105,106,107,108,109,110,111,112,113,114,115,116,117,118,119,120,121,122,123,124,125,126,127,128,129,130]. The European Region (EURO) accounted for seven studies (n = 7) [35,133,134,135,136,138,139], while the Western Pacific Region (WPRO) included three studies (n = 3) [35,131,132]. The African Region (AFRO) and the South-East Asia Region (SEARO) each contributed one study (n = 1) [137,140], and no studies were identified in the Eastern Mediterranean Region (EMRO; n = 0). This distribution refers exclusively to the studies included in this review and does not represent the global distribution of research or policies related to the topic.

### 6.3. Temporal Distribution of Publications

As shown in Figure 3, the temporal distribution of the included studies (n = 104) spans the period from 1999 to 2025, covering more than two decades of scientific production. The earliest study was published in 1999 [56], while the most recent records date from 2025 [44,117,140].

The initial phase (1999–2013) is characterized by low publication density and high temporal dispersion, with only two studies published in 2009 [62,82] and four studies identified between 2010 and 2013 [68,90,115,128], reflecting an incipient stage of academic engagement with the topic. Between 2014 and 2016, a modest increase in publication frequency is observed, with three studies in 2014 [64,70,95], four in 2015 [46,53,65,114], and four in 2016 [39,50,58,134], suggesting a gradual expansion of research interest.

The period from 2017 to 2021 represents a phase of consolidation and progressive growth, with six studies published in 2017, seven in 2018, and eight in 2019, followed by a slight fluctuation with five studies in 2020 and seven in 2021. This phase indicates a transition from sporadic production toward a more stable and continuous research pattern.

A substantial increase in publication volume is observed from 2022 onwards, with fourteen studies published in 2022 [35,45,47,48,51,71,77,98,100,116,123,129,132,133], sixteen in 2023 [43,49,55,61,72,87,88,97,102,111,112,120,121,122,136,137], and a peak of twenty studies in 2024 [37,42,52,54,69,74,84,86,89,91,92,93,99,103,104,118,126,127,131,135]. This upward trend is followed by a decrease to three studies in 2025 [44,117,140], which likely reflects partial year indexing rather than a decline in research interest.

When grouped into temporal strata, the corpus shows a clear concentration of publications in the most recent period (2022–2025), comprising 53 studies, corresponding to the phase of greatest research expansion. This is followed by the period from 2017 to 2021, with 33 studies, representing consolidation and growth. In contrast, earlier publications (1999–2016) account for 18 studies and reflect a comparatively incipient stage, characterized by lower volume and greater dispersion of research output.

### 6.4. Methodological Profile of Included Studies

As shown in Figure 4, the methodological profile of the included studies (n = 104) encompasses quantitative, qualitative, mixed-methods, and review-based designs. Quantitative studies accounted for the largest proportion within the dataset, comprising 37 studies (35.6%) [37,41,46,47,48,54,55,57,60,64,67,68,69,71,73,74,77,81,89,90,93,94,97,101,102,107,108,109,110,112,114,115,121,127,137,138], including cross-sectional designs, longitudinal analyses, quasi-experimental pretest/posttest designs, prospective observational studies, randomized controlled trials, and statistical evaluations based on numerical data. Qualitative studies comprised 24 studies (23.1%) [43,49,56,58,63,79,80,84,100,105,118,119,123,124,125,126,128,129,130,131,132,133,134,139], representing approaches such as content analysis, critical discourse analysis, semi-structured interviews, focus groups, grounded theory, qualitative descriptive designs, narrative interviews, policy analysis, and experience reports. Review-based studies, including systematic reviews, scoping reviews, narrative reviews, literature reviews, knowledge syntheses, commentaries, and conceptual or practice-oriented reviews, accounted for 24 studies (23.1%) [35,36,38,39,40,42,50,52,61,62,70,72,88,95,96,97,98,106,111,113,116,120,135,136,140]. Finally, mixed-methods studies were identified in 19 studies (18.3%) [44,45,51,53,59,65,66,75,76,78,82,83,85,87,91,99,103,117,122], combining qualitative and quantitative approaches through triangulation designs, program evaluations, implementation protocols, survey-interview combinations, and integrated analytical frameworks. These data correspond exclusively to the methodological classification of the 104 studies included in this scoping review.

### 6.5. Thematic Synthesis of Evidence

The selection process was conducted with the aid of the Rayyan tool and took place in three stages. In the first stage, articles were selected based on title reading. In the second stage, abstracts were reviewed. In the third stage, full-text reading was performed for final inclusion. All stages aimed to identify articles that could answer the research question.

The analytical data extracted from the included studies (n = 104) were organized into six main thematic categories, representing the principal empirical patterns identified in the literature (Table 1). The thematic distribution reflects recurrent areas of investigation concerning healthcare training, workforce composition, structural determinants, and lived experiences of marginalized populations, particularly SGM individuals and Black communities.

**Table 1:** Thematic synthesis of included studies on health education for Black populations and SGM.

Thematic Category	Population	Type of Evidence	Key Findings	Studies
Gender-affirming care & clinical competence	SGM individuals, medical/nursing trainees	Program evaluations, observational studies, qualitative analysis	Increased trainee knowledge, comfort, and self-efficacy post-intervention; identified persistent educational gaps regarding hormone therapy and transition management; barriers to shared decision-making reported in clinical settings.	[48,76,77,99,101,119]
Workforce diversity & academic representation	Black providers, trainees, medical faculty	Database reviews, policy analyses, retrospective statistical analyses	Persistent underrepresentation in the physician workforce; lower 10-year promotion rates and retention for Black faculty compared to White peers; projected workforce loss linked to HBCU closures.	[37,41,43,46,113,116]
Social determinants of health (SDOH) & health equity	Black and SGM populations, healthcare trainees	Needs assessments, surveys, cross-sectional studies	High resident confidence contrasted with low actual knowledge of underserved populations; structural racism, housing, poverty, and other social determinants consistently identified as primary disparity drivers.	[49,88,89,93,94,97,115]
Minority stress, stigma, & mental health	SGM youth/adults, Black medical students	Longitudinal studies, surveys, grounded theory, clinical and educational analyses	Distinct psychological distress profiles were identified, including depression, anxiety, suicidality, gender dysphoria, and vulnerability associated with minority stress; rural SGM populations exhibited higher depression vulnerability linked to stigma and barriers to care.	[58,64,69,108,109,110]
Discrimination & intersectionality	Intersectional students, Black, female, SGM individuals, marginalized patients	Multicenter cross-sectional studies, mixed-methods, policy and clinical analyses	Higher statistical probability of explicit discriminatory experiences among marginalized students; clinical reports of disrespectful care, race-based trauma, and unaddressed ethnic differences in clinical assessment practices.	[44,100,127,139]
Inclusive communication & cultural humility	EDI leaders, multidisciplinary trainees, healthcare professionals	Lexicon development, educational simulations, literature reviews, qualitative and mixed-methods studies	Establishment of standard EDI lexicons for mitigation strategies; significant increase in affirmative practice scores post-simulation; widespread need for neutral terminology, correct pronouns, cultural humility, and trauma-informed interactions.	[52,81,83,98,112,118]

Regarding clinical education and practice, the literature demonstrated a consistent focus on gender-affirming care and clinical competence, indicating that targeted educational interventions were associated with increases in trainee knowledge, comfort, and self-efficacy. Despite these gains, the findings also documented persistent gaps in specific areas, including hormone therapy management and transition-related care, as well as reported barriers to shared decision-making in clinical settings [48,76,77,99,101,119].

Closely aligned with this domain, the theme of inclusive communication and cultural humility encompassed studies addressing the development of standardized Equity, Diversity, and Inclusion (EDI) lexicons and educational simulations, with evidence indicating measurable improvements in affirmative practice scores and the identification of ongoing needs related to neutral terminology, correct pronoun use, cultural humility, and trauma-informed communication [52,81,83,98,112,118].

A second major thematic cluster addressed workforce diversity and academic representation among Black healthcare providers, trainees, and faculty members. Within this cluster, the included studies generally treated Black professionals as a single aggregate category, without disaggregating findings by gender identity, sexual orientation, class, or other axes of social differentiation. The evidence consistently documented persistent underrepresentation within the physician workforce, alongside disparities in long-term promotion and retention, including lower ten-year advancement rates among Black faculty compared with their White peers, as well as projected workforce reductions associated with the closure of historically Black colleges and universities (HBCUs) [37,41,43,46,113,116]. This aggregation, while reflecting how the source studies operationalized race, obscures the differentiated positions occupied by Black women, Black professionals with diverse sexual orientations and gender identities, and others situated at the intersections of multiple systems of inequality.

Across these studies, structural factors such as racism, housing instability, socioeconomic deprivation, and other social determinants were consistently identified as central drivers of health disparities [49,88,89,93,94,97,115]. Finally, the literature also encompassed themes related to minority stress, stigma, and mental health, as well as discrimination and intersectionality, capturing the experiential dimensions of marginalized populations within healthcare and educational environments. The evidence documented distinct patterns of psychological distress, including depression, anxiety, suicidality, gender dysphoria, and vulnerability associated with minority stress and stigma, particularly among sexual and gender minority populations and Black medical students [58,64,69,108,109,110]. In parallel, studies addressing discrimination and intersectionality reported higher probabilities of explicit discriminatory experiences among individuals with overlapping marginalized identities, including documented cases of disrespectful care, race-based trauma, and inadequately addressed ethnic differences in clinical assessment practices [44,100,127,139].

## 7. Discussion

Across the included studies, educational interventions are predominantly oriented toward short-term outcomes, particularly the enhancement of knowledge, perceived comfort, and self-efficacy in relation to gender-affirming care and culturally responsive practices, frequently operationalized through workshops and simulation-based strategies. In parallel, the literature consistently documents the underrepresentation of Black professionals within healthcare systems and academic institutions, accompanied by persistent structural constraints affecting career progression and retention. Another recurring pattern lies in the centrality attributed to social determinants of health, with structural racism, housing conditions, and socioeconomic inequality systematically identified as key factors shaping health disparities among Black and SGM populations. These elements appear not as peripheral considerations but as core dimensions of the empirical evidence, reinforcing their relevance within health education contexts.

A substantial convergence emerges in the reported effectiveness of targeted educational interventions in improving self-reported measures such as confidence, comfort, and theoretical understanding among trainees. However, this apparent consensus is accompanied by a notable divergence regarding the extent to which these gains translate into measurable clinical competence or sustained changes in professional practice. While part of the literature emphasizes the importance of structured, competency-based curricula, other studies indicate that learning related to minority health frequently occurs in an unstructured manner within clinical environments, often generating uncertainty among trainees due to insufficient formal preparation and limited faculty expertise. This tension between formal curricular design and experiential learning highlights an unresolved dimension in the training process.

The distribution of evidence reveals a pronounced concentration of studies in North American and other high-income settings, with limited representation of low- and middle-income contexts, thereby restricting the global scope of current knowledge on health education practices. This asymmetry is not accidental. In many underrepresented regions, institutional criminalization and the lack of legal recognition for SGM populations hinder both the implementation of inclusive health curricula and the conduct of safe scientific research. These legal, political, and institutional barriers perpetuate invisibility, constrain the production of locally grounded evidence, and reinforce neglect within health professions education.

Although the review imposed no geographical restrictions, this should not be read as assuming equivalence across countries. Health education systems are shaped by historically specific configurations of colonialism, racialization, gender regulation, and sexuality-related norms. Thus, while studies from colonially shaped contexts may share concerns regarding the marginalization of Black populations, women, and SGM communities, their findings must be interpreted in light of distinct racial classifications, legal frameworks, health systems, curricular models, and sociocultural meanings of race, gender, and sexuality.

In addition, there is a scarcity of investigations examining long-term outcomes, particularly those related to patient-level impacts and the durability of acquired competencies over time. Another notable gap concerns the limited integration of analytical approaches that simultaneously address the health needs of Black and SGM populations. Most studies treat these groups as distinct categories, with relatively few exploring the intersections between race, gender identity, and sexuality in educational contexts.

This separation is not exclusive to educational literature. A rapid scoping review of Brazilian scientific production on LGBTQIA+ health found no studies that incorporated race, skin color, or social class into their analyses, suggesting that the parallel treatment of these dimensions extends beyond educational research and characterizes the broader field of health research [141]. The scarcity of intersectional evidence may therefore stem, in part, from the architecture of data systems and research agendas that shape the field, rather than from the absence of the phenomenon itself.

The methodological landscape is largely characterized by cross-sectional quantitative designs, qualitative descriptive analyses, and short-term pre- and post-intervention studies. The relative absence of longitudinal approaches restricts the ability to assess sustained changes in professional behavior and limits the evaluation of long-term educational impact. Furthermore, a significant proportion of the studies relies on single-institution settings and self-reported data, which constrains external validity and reinforces a reliance on subjective indicators of competence. Objective measures of clinical performance and multi-institutional designs remain comparatively underrepresented within the dataset.

The mapped evidence indicates that, although diversity-related themes have gained visibility within health education, their incorporation into formal curricula remains fragmented and frequently non-mandatory. The engagement with intersectionality as a theoretical framework is present but uneven, with limited translation into structured pedagogical tools capable of integrating race, gender, sexuality, and class within competency-based training. As a result, the literature addresses the review objective in a partial manner, offering insight into existing initiatives while simultaneously revealing discontinuities in their curricular implementation.

The synthesis highlights the absence of standardized curricular models that move beyond introductory approaches to cultural competence and engage directly with structural forms of inequality, including racism and institutionalized discrimination related to gender and sexuality. There is also a lack of evidence on pedagogical strategies explicitly designed to operationalize intersectionality within professional training, particularly in ways that enable practitioners to recognize and respond to overlapping axes of vulnerability. In addition, the literature provides limited attention to the training environment itself, with few studies examining structural interventions aimed at addressing minority stress, unequal academic trajectories, and discriminatory experiences among Black and SGM trainees.

## 8. Conclusions

The findings of this scoping review indicate that the literature addressing the health of Black and SGM populations within health education has expanded in recent years, with a strong concentration of studies in specific thematic domains, particularly those related to gender-affirming care, social determinants of health, and diversity-oriented educational interventions. Despite this expansion, the available evidence remains characterized by uneven thematic distribution and a predominance of studies focused on short-term educational outcomes and localized training initiatives.

The evidence shows that educational interventions frequently emphasize improvements in knowledge, self-reported competence, and perceived preparedness among healthcare trainees. However, these interventions are predominantly implemented as isolated or program-specific strategies, rather than as components of systematically integrated curricula. As a result, the incorporation of structural dimensions such as racism, stigma, and intersectionality into health education remains variable and often limited in scope, reflecting differences in institutional priorities and educational models and contexts.

In addition to the concentration of themes, the review highlights important gaps related to the continuity and impact of educational strategies. The predominance of cross-sectional designs, short-term evaluations, and single-institution studies limits the ability to assess whether improvements in training translate into sustained changes in clinical practice or into measurable effects on patient outcomes. Furthermore, the limited representation of studies from regions outside North America restricts the global scope of the evidence, as most included studies were conducted in the United States and Canada.

The review also identifies gaps in the integration of intersectional approaches within health education. Although several studies address race, gender identity, and sexual orientation as distinct analytical dimensions, fewer studies examine their combined effects within training environments. In addition, limited attention has been given to structural conditions affecting healthcare trainees themselves, including discrimination, minority stress, and barriers to academic progression.

Future research should prioritize the development of longitudinal and multi-institutional studies capable of evaluating the sustained impact of educational interventions on both professional practice and health outcomes. Expanding the geographical diversity of studies and incorporating intersectional analytical frameworks may contribute to a more comprehensive understanding of how health education can address the needs of historically marginalized populations.

Overall, the findings indicate that, although diversity-related content is increasingly present in health education, the current body of literature reflects a field that remains focused on short-term, context-specific interventions, with limited capacity to capture long-term, structural, and intersectional dimensions of health training and practice.

## Data Availability

No new data were created or analyzed in this study.

## Supplementary Materials

The following supporting information can be downloaded at: https://www.mdpi.com/article/10.3390/nursrep16070231/s1; Supplementary Material SI: Search strategy; Supplementary Material SII: Data Extraction Instrument; Supplementary Material SIII: Thematic gap matrix by region and level of education.

## Public Involvement Statement

No public involvement in any aspect of this research.

## Guidelines and Standards Statement

This manuscript was drafted against the JBI Methodology for Scoping Reviews (https://jbi-global-wiki.refined.site/space/MANUAL/355862497/10.+Scoping+reviews, accessed on 7 May 2025) and the PRISMA extension for Scoping Reviews (https://www.equator-network.org/reporting-guidelines/prisma-scr/, accessed on 7 May 2025).

## Use of Artificial Intelligence

This study involved the use of generative artificial intelligence tools as operational support in specific stages of the scoping review, without replacing the role of the researchers in analytical and decision-making processes. ChatGPT (paid version) was used to assist in the development and refinement of structured prompts, aiming to standardize procedures such as data extraction, initial categorization, and the organization of synthesis tables, always based on criteria previously defined in the review protocol. NotebookLM (paid version) was employed to support the organization and assisted reading of the included studies, facilitating the identification of information according to the predefined data extraction instrument. The use of this tool was restricted to supporting the retrieval and structuring of information, without delegating interpretative or analytical functions. In addition, generative AI tools were used to support the creation of visual materials. Empirical data produced by the researchers were used to generate structured prompts, which were then applied in an image generation system (Gemini 3.5 Flash) to produce figures that represent the results of the review. All visual outputs were based on real data and were reviewed and validated by the researchers prior to inclusion in the manuscript. All tools were used in their paid versions, with data sharing or training features disabled, in order to preserve confidentiality and data protection. The researchers maintained full responsibility for the accuracy, integrity, and interpretation of all content presented in this study.

## Abbreviations

The following abbreviations are used in this manuscript:

LGBTQIA+	lesbian, gay, bisexual, transgender, queer or questioning, intersex, asexual, and more
PEH	Permanent Education in Health
JBI	A global organization promoting and supporting evidence-based decisions that improve health and health service delivery.
PRISMA	Preferred Reporting Items for Systematic reviews and Meta-Analyses
PRISMA-ScR	PRISMA Extension for Scoping Reviews
BVS/VHL	Biblioteca Virtual em Saúde/Virtual Health Library
BDTD	Brazilian Digital Library of Theses and Dissertations
NDLTD	Networked Digital Library of Theses and Dissertations
PQDT	ProQuest Dissertations & Theses Global
NLM	National Library of Medicine
NIH/NLM	National Library of Medicine/National Institutes of Health
AFRO	African Region
AMRO	Region of the Americas
EMRO	Eastern Mediterranean Region
EURO	European Region
SEARO	South-East Asia Region
SGM	Sexual and Gender Minorities
WPRO	Western Pacific Region
