# Supplementary material for: Health of Black Populations and Sexual and Gender Minorities in Health Education: A Scoping Review
Source: Nurs Rep. 2026 Jul 2;16(7):231. doi: 10.3390/nursrep16070231 (PMC13415247; doi:10.3390/nursrep16070231)
Supplement: Supplementary file 1 [file nursrep-16-00231-s001.zip › nursrep-4294451-supplementary.pdf]

Supplementary Material SI: Search strategy

13 databases/Information sources include Scopus (Elsevier), Web of Science (Clarivate), MEDLINE (Ovid), PubMed (National Library of Medicine – NLM) , PubMed PMC (National Library of Medicine/National Institutes of Health – NIH/NLM), Embase (Elsevier), Virtual Health Library – VHL/Biblioteca Vitual em Saúde (BIREME/OPAS/OMS), CINAHL (EBSCOhost), ERIC (EBSCOhost), Cochrane (Wiley) and grey literature include Brazilian Digital Library of Theses and Dissertations – BDTD (Instituto Brasileiro de Informação em Ciência e Tecnologia – IBICT), Networked Digital Library of Theses and Dissertations – NDLTD and the ProQuest Dissertations & Theses Global – PQDT (Clarivate).

Search carried out: 07/05/2025

SCOPUS (Elsevier)

SEARCH	QUERY	RECORDS RETRIEVED
#1	(TITLE-ABS-KEY ("Black People" OR "Black Peoples" OR "People, Black" OR "Black Person" OR "Black Persons" OR "Negroid Race" OR "Negroid Races" OR "Race, Negroid" OR "African Continental Ancestry Group") OR TITLE-ABS-KEY ("Black Population" OR "Black man" OR "Black race" OR negroid OR "Negroid race" OR negroids) OR TITLE-ABS-KEY ("Black or African American" OR "Black Americans" OR "American, Black" OR "Black American" OR blacks OR negroes OR negro OR "African Americans" OR "African American" OR "American, African" OR "Afro-American" OR "Afro American" OR "Afro-Americans" OR "Afro Americans" OR "African-Americans" OR "African-American") OR TITLE-ABS-KEY ("American blacks" OR "American Negro" OR "black American" OR "black or African American"))	853.640
#2	(TITLE-ABS-KEY ("Sexual and Gender Minorities" OR "LGBT Person" OR "Persons, LGBT" OR "LGBTQ Person" OR "Person, LGBTQ" OR "Persons, LGBTQ" OR "Non-Heterosexual Persons" OR "Non Heterosexual Persons" OR "LBG Persons" OR "Sexual Minorities" OR "Minorities, Sexual" OR "Minority, Sexual" OR "Sexual Minority" OR "Non-Heterosexuals" OR "Non Heterosexuals" OR "Non-Heterosexual" OR "Sexual Dissidents" OR "Sexual Dissident" OR "GLBT Persons" OR "GLBT Person" OR gays OR gay OR "Men Who Have Sex With Men" OR lesbians OR lesbian OR "Women Who Have Sex With Women" OR homosexuals OR homosexual) OR TITLE-ABS-KEY ("sexual and gender minority") OR TITLE-ABS-KEY ("LGBTQIA+ people" OR pansexual OR questioning OR " transgender and intersex" OR transgender OR "GLBTI+" OR "GLBTQ+" OR "LGBTI+" OR "LGBTIQ+" OR "LGBTIQA+" OR "LGBTIQQ" OR "LGBTQ people" OR "LGBTQ+" OR "LGBTQ2" OR "LGBTQ2S" OR "LGBTQ2SIA+" OR "LGBTQA" OR "LGBTQAI" OR "LGBTQIA" OR "LGBTQIA+" OR "LGBTQIA2S+" OR "LGBTQQ" OR "LGBTQQIA" OR "LGBTTQ+" OR "LGTBQA" OR asexual OR "two-spirit" OR "2-spirit" OR "2 spirit") OR TITLE-ABS-KEY (lgbtqiapn+) OR TITLE-ABS-KEY (homosexuality OR "Ego-Dystonic Homosexuality") OR TITLE-ABS-KEY ("Homosexuality, Female" OR "Female Homosexuality" OR lesbianism) OR TITLE-ABS-KEY ("Homosexuality, Male" OR "Male Homosexuality") OR TITLE-ABS-KEY ("gender nonbinary" OR "gender non binary" OR "gender non conforming" OR "gender nonconforming" OR "non binary AFAB" OR "non binary gender" OR "non binary gender identity" OR "non binary individuals" OR "non binary people" OR "non conforming gender" OR "nonbinary AFAB" OR "nonbinary gender" OR "nonbinary individuals" OR "nonbinary people") OR TITLE-ABS-KEY ("gender expansive youth" OR "gender-expansive people" OR "gender-fluid" OR "gender-queer" OR "gender-questioning" OR "genderexpansive" OR genderfluid OR genderqueer OR genderquestioning OR "TGNB people" OR "TNB individuals" OR "TNB people" OR "transgender and gender non-	238.488

	conforming" OR "transgender and gender nonconforming" OR "transgender and nonbinary") OR TITLE-ABS-KEY ("Gender Identity"))	
#3	**#1 OR #2**	**1,081.480**
#4	(TITLE-ABS-KEY ("Vulnerable Populations" OR "Vulnerable Population" OR "Disadvantaged Populations" OR "Disadvantaged Population" OR "Sensitive Populations" OR "Sensitive Population" OR "Sensitive Population Groups" OR "Sensitive Population Group" OR "Underserved Population" OR "Underserved Populations") OR TITLE-ABS-KEY ("vulnerable minorities" OR "vulnerable minority" OR "vulnerable minority population" OR "vulnerable people" OR "vulnerable person" OR "vulnerable persons") OR TITLE-ABS-KEY ("Social Vulnerability" OR "Social Vulnerabilities") OR TITLE-ABS-KEY (vulnerability OR vulnerable))	626.888
#5	(TITLE-ABS-KEY ("Health Inequities" OR "Health Inequity" OR "Health Inequalities" OR "Health Inequality" OR "Health Disparities" OR "Health Disparity") OR TITLE-ABS-KEY ("disparity in health" OR "health status disparities" OR "health status disparity" OR "inequality in health" OR "inequity in health" OR "socioeconomic disparities in health"))	91.579
#6	(TITLE-ABS-KEY (health) AND TITLE-ABS-KEY (inequities OR inequity OR inequalities OR inequality OR disparities OR disparity))	205.311
#7	**#5 OR #6**	**205.311**
#8	**#4 AND #7**	**18.438**
#9	(TITLE-ABS-KEY (teaching OR "Training Techniques" OR "Training Technique" OR "Training Technics" OR "Training Technic" OR pedagogy OR pedagogies OR "Teaching Methods" OR "Teaching Method" OR "Academic Training" OR "Training Activities" OR "Training Activity" OR "Educational Techniques" OR "Educational Technique" OR "Educational Technics" OR "Educational Technic") OR TITLE-ABS-KEY (curriculum OR curricula OR "Short-Term Courses" OR "Short Term Courses") OR TITLE-ABS-KEY ("competency-based education" OR "integrated curriculum") OR TITLE-ABS-KEY ("Education, Continuing" OR "Continuous Learning" OR "Lifelong Learning" OR "Life-Long Learning" OR "Life Long Learning" "Continuing Education") OR TITLE-ABS-KEY ("Education, Professional" OR "Professional Education") OR TITLE-ABS-KEY ("Interprofessional Education" OR "Education, Interprofessional") OR TITLE-ABS-KEY ("inter-professional education" OR "cross training" OR multi-skilling OR multiskilling) OR TITLE-ABS-KEY ("Professional Training") OR TITLE-ABS-KEY (schools OR school OR "Secondary School" OR "Secondary Schools") OR TITLE-ABS-KEY ("Health Personnel" OR "Healthcare Workers" OR "Healthcare Worker" OR "Health Care Providers" OR "Health Care Provider" OR "Healthcare Providers" OR "Healthcare Provider" OR "Health Care Professionals" OR "Health Care Professional"))	2,887.124
#10	**#3 AND #8 AND #8**	**473**

WEB OF SCIENCE (Clarivate)

SEARCH	QUERY	RECORDS RETRIEVE D
#1	"Black People" OR "Black Peoples" OR "People, Black" OR "Black Person" OR "Black Persons" OR "Negroid Race" OR "Negroid Races" OR "Race, Negroid" OR "African Continental Ancestry Group" (Topic) or "Black Population" OR "Black man" OR "Black race" OR Negroid OR "Negroid race" OR Negroids (Topic) or "Black or African American" OR "Black Americans" OR "American, Black" OR "Black American" OR Blacks OR Negroes OR Negro OR "African Americans" OR "African American" OR "American, African" OR "Afro-American" OR "Afro American" OR "Afro-Americans" OR "Afro Americans" OR "African-Americans" OR "African-American" (Topic) or "American blacks" OR "American Negro" OR "black American" OR "black or African American" (Topic)	817.025

#2	"Sexual and Gender Minorities" OR "LGBT Person" OR "Persons, LGBT" OR "LGBTQ Person" OR "Person, LGBTQ" OR "Persons, LGBTQ" OR "Non-Heterosexual Persons" OR "Non Heterosexual Persons" OR "LBG Persons" OR "Sexual Minorities" OR "Minorities, Sexual" OR "Minority, Sexual" OR "Sexual Minority" OR "Non-Heterosexuals" OR "Non Heterosexuals" OR "Non-Heterosexual" OR "Sexual Dissidents" OR "Sexual Dissident" OR "GLBT Persons" OR "GLBT Person" OR Gays OR Gay OR "Men Who Have Sex With Men" OR Lesbians OR Lesbian OR "Women Who Have Sex With Women" OR Homosexuals OR Homosexual (Topic) or "sexual and gender minority" (Topic) or "LGBTQIA+ people" OR pansexual OR questioning OR " transgender and intersex" OR transgender OR "GLBTI+" OR "GLBTQ+" OR "LGBTI+" OR "LGBTIQ+" OR "LGBTIQA+" OR "LGBTIQQ" OR "LGBTQ people" OR "LGBTQ+" OR "LGBTQ2" OR "LGBTQ2S" OR "LGBTQ2SIA+" OR "LGBTQA" OR "LGBTQAI" OR "LGBTQIA" OR "LGBTQIA+" OR "LGBTQIA2S+" OR "LGBTQQ" OR "LGBTQQIA" OR "LGBTTQ+" OR "LGTBQA" OR asexual OR "two-spirit" OR "2-spirit" OR "2 spirit" (Topic) or LGBTQIAPN+ (Topic) or Homosexuality OR "Ego-Dystonic Homosexuality" (Topic) or "Homosexuality, Female" OR "Female Homosexuality" OR Lesbianism (Topic) or "Homosexuality, Male" OR "Male Homosexuality" (Topic) or "gender nonbinary" OR "gender non binary" OR "gender non conforming" OR "gender nonconforming" OR "non binary AFAB" OR "non binary gender" OR "non binary gender identity" OR "non binary individuals" OR "non binary people" OR "non conforming gender" OR "nonbinary AFAB" OR "nonbinary gender" OR "nonbinary individuals" OR "nonbinary people" (Topic) or "gender expansive youth" OR "gender-expansive people" OR "gender-fluid" OR "gender-queer" OR "gender-questioning" OR "genderexpansive" OR genderfluid OR genderqueer OR genderquestioning OR "TGNB people" OR "TNB individuals" OR "TNB people" OR "transgender and gender non-conforming" OR "transgender and gender nonconforming" OR "transgender and nonbinary" (Topic) or "Gender Identity" (Topic)	1.593.313
#3	**#1 OR #2**	**2,112,550**
#4	"Vulnerable Populations" OR "Vulnerable Population" OR "Disadvantaged Populations" OR "Disadvantaged Population" OR "Sensitive Populations" OR "Sensitive Population" OR "Sensitive Population Groups" OR "Sensitive Population Group" OR "Underserved Population" OR "Underserved Populations" (Topic) or "Vulnerable Populations" OR "Vulnerable Population" OR "Disadvantaged Populations" OR "Disadvantaged Population" OR "Sensitive Populations" OR "Sensitive Population" OR "Sensitive Population Groups" OR "Sensitive Population Group" OR "Underserved Population" OR "Underserved Populations" (Topic) or "vulnerable minorities" OR "vulnerable minority" OR "vulnerable minority population" OR "vulnerable people" OR "vulnerable person" OR "vulnerable persons" (Topic) or "vulnerable minorities" OR "vulnerable minority" OR "vulnerable minority population" OR "vulnerable people" OR "vulnerable person" OR "vulnerable persons" (Topic) or "Social Vulnerability" OR "Social Vulnerabilities" (Topic) or Vulnerability OR Vulnerable (Topic)	513.956
#5	"Health Inequities" OR "Health Inequity" OR "Health Inequalities" OR "Health Inequality" OR "Health Disparities" OR "Health Disparity" (Topic) or "disparity in health" OR "health status disparities" OR "health status disparity" OR "inequality in health" OR "inequity in health" OR "socioeconomic disparities in health" (Topic)	54.187
#6	health (Topic) and Inequities OR Inequity OR Inequalities OR Inequality OR Disparities OR Disparity (Topic)	176.509
#7	**#5 OR #6**	176.509
#8	**#4 AND #7**	15.491
#9	((((((((TS=(Teaching OR "Training Techniques" OR "Training Technique" OR "Training Technics" OR "Training Technic" OR Pedagogy OR Pedagogies OR "Teaching Methods" OR "Teaching Method" OR "Academic Training" OR "Training Activities" OR "Training Activity" OR "Educational Techniques" OR "Educational Technique" OR "Educational Technics" OR "Educational Technic")) OR TS=(Curriculum OR Curricula OR "Short-Term Courses" OR "Short Term Courses")) OR TS=("competency-based education" OR "integrated curriculum")) OR TS=("Education, Continuing"	1.870.267

	OR "Continuous Learning" OR "Lifelong Learning" OR "Life-Long Learning" OR "Life Long Learning" "Continuing Education")) OR TS=("Education, Professional" OR "Professional Education")) OR TS=("Interprofessional Education" OR "Education, Interprofessional")) OR TS=("inter-professional education" OR "cross training" OR multi-skilling OR multiskilling)) OR TS=("Professional Training")) OR TS=(Schools OR School OR "Secondary School" OR "Secondary Schools")) OR TS=("Health Personnel" OR "Healthcare Workers" OR "Healthcare Worker" OR "Health Care Providers" OR "Health Care Provider" OR "Healthcare Providers" OR "Healthcare Provider" OR "Health Care Professionals" OR "Health Care Professional")	
#10	**#3 AND #8 AND #9**	**514**

MEDLINE (OVID)

SEARCH	QUERY	RECORDS RETRIEVED
#1	exp *Black People/ OR ("Black People" or "Black Peoples" or "People, Black" or "Black Person" or "Black Persons" or "Negroid Race" or "Negroid Races" or "Race, Negroid" or "African Continental Ancestry Group").ab. or ("Black People" or "Black Peoples" or "People, Black" or "Black Person" or "Black Persons" or "Negroid Race" or "Negroid Races" or "Race, Negroid" or "African Continental Ancestry Group").ti. or ("Black Population" or "Black man" or "Black race" or Negroid or "Negroid race" or Negroids).ab. or ("Black Population" or "Black man" or "Black race" or Negroid or "Negroid race" or Negroids).ti. OR ("Black or African American" or "Black Americans" or "American, Black" or "Black American" or Blacks or Negroes or Negro or "African Americans" or "African American" or "American, African" or "Afro-American" or "Afro American" or "Afro-Americans" or "Afro Americans" or "African-Americans" or "African-American").ab. or ("Black or African American" or "Black Americans" or "American, Black" or "Black American" or Blacks or Negroes or Negro or "African Americans" or "African American" or "American, African" or "Afro-American" or "Afro American" or "Afro-Americans" or "Afro Americans" or "African-Americans" or "African-American").ti. or ("American blacks" or "American Negro" or "black American" or "black or African American").ab. or ("American blacks" or "American Negro" or "black American" or "black or African American").ti.	113.483
#2	("Sexual and Gender Minorities" or "LGBT Person" or "Persons, LGBT" or "LGBTQ Person" or "Person, LGBTQ" or "Persons, LGBTQ" or "Non-Heterosexual Persons" or "Non Heterosexual Persons" or "LBG Persons" or "Sexual Minorities" or "Minorities, Sexual" or "Minority, Sexual" or "Sexual Minority" or "Non-Heterosexuals" or "Non Heterosexuals" or "Non-Heterosexual" or "Sexual Dissidents" or "Sexual Dissident" or "GLBT Persons" or "GLBT Person" or Gays or Gay or "Men Who Have Sex With Men" or Lesbians or Lesbian or "Women Who Have Sex With Women" or Homosexuals or Homosexual).ab. or ("Sexual and Gender Minorities" or "LGBT Person" or "Persons, LGBT" or "LGBTQ Person" or "Person, LGBTQ" or "Persons, LGBTQ" or "Non-Heterosexual Persons" or "Non Heterosexual Persons" or "LBG Persons" or "Sexual Minorities" or "Minorities, Sexual" or "Minority, Sexual" or "Sexual Minority" or "Non-Heterosexuals" or "Non Heterosexuals" or "Non-Heterosexual" or "Sexual Dissidents" or "Sexual Dissident" or "GLBT Persons" or "GLBT Person" or Gays or Gay or "Men Who Have Sex With Men" or Lesbians or Lesbian or "Women Who Have Sex With Women" or Homosexuals or Homosexual).ti. or "sexual and gender minority".ab. or "sexual and gender minority".ti. OR ("LGBTQIA+ people" or pansexual or questioning or " transgender and intersex" or transgender or "GLBTI+" or "GLBTQ+" or "LGBTI+" or "LGBTIQ+" or "LGBTIQA+" or "LGBTIQQ" or "LGBTQ people" or "LGBTQ+" or "LGBTQ2" or "LGBTQ2S" or "LGBTQ2SIA+" or "LGBTQA" or "LGBTQAI" or "LGBTQIA" or "LGBTQIA+" or "LGBTQIA2S+" or "LGBTQQ" or "LGBTQQIA" or "LGBTTQ+" or "LGTBQA" or asexual or "two-spirit" or "2-spirit" or "2 spirit").ab. or ("LGBTQIA+ people" or pansexual or questioning or " transgender and intersex" or transgender or "GLBTI+" or "GLBTQ+" or "LGBTI+"	87.815

	or "LGBTIQ+" or "LGBTIQA+" or "LGBTIQQ" or "LGBTQ people" or "LGBTQ+" or "LGBTQ2" or "LGBTQ2S" or "LGBTQ2SIA+" or "LGBTQA" or "LGBTQAI" or "LGBTQIA" or "LGBTQIA+" or "LGBTQIA2S+" or "LGBTQQ" or "LGBTQQIA" or "LGBTTQ+" or "LGTBQA" or asexual or "two-spirit" or "2-spirit" or "2 spirit").ti. or LGBTQIAPN+.ab. or LGBTQIAPN+.ti. OR exp *Homosexuality/ OR (Homosexuality or "Ego-Dystonic Homosexuality").ab. or (Homosexuality or "Ego-Dystonic Homosexuality").ti. OR exp *Homosexuality, Female/ OR ("Homosexuality, Female" or "Female Homosexuality" or Lesbianism).ab. or ("Homosexuality, Female" or "Female Homosexuality" or Lesbianism).ti. OR exp *Homosexuality, Male/ OR ("Homosexuality, Male" or "Male Homosexuality").ab. or ("Homosexuality, Male" or "Male Homosexuality").ti. OR ("gender nonbinary" or "gender non binary" or "gender non conforming" or "gender nonconforming" or "non binary AFAB" or "non binary gender" or "non binary gender identity" or "non binary individuals" or "non binary people" or "non conforming gender" or "nonbinary AFAB" or "nonbinary gender" or "nonbinary individuals" or "nonbinary people").ab. or ("gender nonbinary" or "gender non binary" or "gender non conforming" or "gender nonconforming" or "non binary AFAB" or "non binary gender" or "non binary gender identity" or "non binary individuals" or "non binary people" or "non conforming gender" or "nonbinary AFAB" or "nonbinary gender" or "nonbinary individuals" or "nonbinary people").ti. or ("gender expansive youth" or "gender-expansive people" or "gender-fluid" or "gender-queer" or "gender-questioning" or "genderexpansive" or genderfluid or genderqueer or genderquestioning or "TGNB people" or "TNB individuals" or "TNB people" or "transgender and gender non-conforming" or "transgender and gender nonconforming" or "transgender and nonbinary").ab. or ("gender expansive youth" or "gender-expansive people" or "gender-fluid" or "gender-queer" or "gender-questioning" or "genderexpansive" or genderfluid or genderqueer or genderquestioning or "TGNB people" or "TNB individuals" or "TNB people" or "transgender and gender non-conforming" or "transgender and gender nonconforming" or "transgender and nonbinary").ti. OR exp *Gender Identity/ OR "Gender Identity".ab. or "Gender Identity".ti.	
#3	**#1 OR #2**	**198.794**
#4	exp *Vulnerable Populations/ OR ("Vulnerable Populations" or "Vulnerable Population" or "Disadvantaged Populations" or "Disadvantaged Population" or "Sensitive Populations" or "Sensitive Population" or "Sensitive Population Groups" or "Sensitive Population Group" or "Underserved Population" or "Underserved Populations").ab. or ("Vulnerable Populations" or "Vulnerable Population" or "Disadvantaged Populations" or "Disadvantaged Population" or "Sensitive Populations" or "Sensitive Population" or "Sensitive Population Groups" or "Sensitive Population Group" or "Underserved Population" or "Underserved Populations").ti. or ("vulnerable minorities" or "vulnerable minority" or "vulnerable minority population" or "vulnerable people" or "vulnerable person" or "vulnerable persons").ab. or ("vulnerable minorities" or "vulnerable minority" or "vulnerable minority population" or "vulnerable people" or "vulnerable person" or "vulnerable persons").ti. OR exp *Social Vulnerability/ OR ("Social Vulnerability" or "Social Vulnerabilities").ab. or ("Social Vulnerability" or "Social Vulnerabilities").ti. or (Vulnerability or Vulnerable).ab. or (Vulnerability or Vulnerable).ti.	181.818
#5	exp *Health Inequities/ OR ("Health Inequities" or "Health Inequity" or "Health Inequalities" or "Health Inequality" or "Health Disparities" or "Health Disparity").ab. or ("Health Inequities" or "Health Inequity" or "Health Inequalities" or "Health Inequality" or "Health Disparities" or "Health Disparity").ti. or ("disparity in health" or "health status disparities" or "health status disparity" or "inequality in health" or "inequity in health" or "socioeconomic disparities in health").ab. or ("disparity in health" or "health status disparities" or "health status disparity" or "inequality in health" or "inequity in health" or "socioeconomic disparities in health").ti.	50.852
#6	health.ab. or health.ti. AND (Inequities or Inequity or Inequalities or Inequality or Disparities or Disparity).ab. or (Inequities or Inequity or Inequalities or Inequality or Disparities or Disparity).ti.	104.028
#7	**#5 OR #6**	**113.466**
#8	**#4 AND #7**	**8.277**

#9	exp *Teaching/ OR (Teaching or "Training Techniques" or "Training Technique" or "Training Technics" or "Training Technic" or Pedagogy or Pedagogies or "Teaching Methods" or "Teaching Method" or "Academic Training" or "Training Activities" or "Training Activity" or "Educational Techniques" or "Educational Technique" or "Educational Technics" or "Educational Technic").ab. or (Teaching or "Training Techniques" or "Training Technique" or "Training Technics" or "Training Technic" or Pedagogy or Pedagogies or "Teaching Methods" or "Teaching Method" or "Academic Training" or "Training Activities" or "Training Activity" or "Educational Techniques" or "Educational Technique" or "Educational Technics" or "Educational Technic").ti. OR exp *Curriculum/ OR (Curriculum or Curricula or "Short-Term Courses" or "Short Term Courses").ab. or (Curriculum or Curricula or "Short-Term Courses" or "Short Term Courses").ti. OR ("competency-based education" or "integrated curriculum").ab. or ("competency-based education" or "integrated curriculum").ti. OR exp *Education, Continuing/ OR ("Education, Continuing" or "Continuous Learning" or "Lifelong Learning" or "Life-Long Learning" or "Life Long Learning Continuing Education").ab. or ("Education, Continuing" or "Continuous Learning" or "Lifelong Learning" or "Life-Long Learning" or "Life Long Learning Continuing Education").ti. OR exp *Education, Professional/ OR ("Education, Professional" or "Professional Education").ab. or ("Education, Professional" or "Professional Education").ti. OR exp *Interprofessional Education/ OR ("Interprofessional Education" or "Education, Interprofessional").ab. or ("Interprofessional Education" or "Education, Interprofessional").ti. or ("inter-professional education" or "cross training" or multi-skilling or multiskilling).ab. or ("inter-professional education" or "cross training" or multi-skilling or multiskilling).ti. OR "Professional Training".ab. or "Professional Training".ti. OR exp *Schools/ OR (Schools or School or "Secondary School" or "Secondary Schools").ab. or (Schools or School or "Secondary School" or "Secondary Schools").ti. OR exp *Health Personnel/ OR ("Health Personnel" or "Healthcare Workers" or "Healthcare Worker" or "Health Care Providers" or "Health Care Provider" or "Healthcare Providers" or "Healthcare Provider" or "Health Care Professionals" or "Health Care Professional").ab. or ("Health Personnel" or "Healthcare Workers" or "Healthcare Worker" or "Health Care Providers" or "Health Care Provider" or "Healthcare Providers" or "Healthcare Provider" or "Health Care Professionals" or "Health Care Professional").ti.	1.206.517
#10	**#3 AND #8 AND #9**	**164**

PUBMED (NLM)

SEARCH	QUERY	RECORDS RETRIEVED
#1	(((Black People[MeSH Terms]) OR ("Black People"[Title/Abstract] OR "Black Peoples"[Title/Abstract] OR "People, Black"[Title/Abstract] OR "Black Person"[Title/Abstract] OR "Black Persons"[Title/Abstract] OR "Negroid Race"[Title/Abstract] OR "Negroid Races"[Title/Abstract] OR "Race, Negroid"[Title/Abstract] OR "African Continental Ancestry Group"[Title/Abstract])) OR ("Black Population"[Title/Abstract] OR "Black man"[Title/Abstract] OR "Black race"[Title/Abstract] OR Negroid[Title/Abstract] OR "Negroid race"[Title/Abstract] OR Negroids[Title/Abstract])) OR (((Black or African American[MeSH Terms]) OR ("Black or African American"[Title/Abstract] OR "Black Americans"[Title/Abstract] OR "American, Black"[Title/Abstract] OR "Black American"[Title/Abstract] OR Blacks[Title/Abstract] OR Negroes[Title/Abstract] OR Negro[Title/Abstract] OR "African Americans"[Title/Abstract] OR "African American"[Title/Abstract] OR "American, African"[Title/Abstract] OR "Afro-American"[Title/Abstract] OR "Afro American"[Title/Abstract] OR "Afro-Americans"[Title/Abstract] OR "Afro Americans"[Title/Abstract] OR "African-Americans"[Title/Abstract] OR "African-American"[Title/Abstract])) OR ("American	342.310

	blacks"[Title/Abstract] OR "American Negro"[Title/Abstract] OR "black American"[Title/Abstract] OR "black or African American"[Title/Abstract]))	
#2	((((((Sexual and Gender Minorities[MeSH Terms]) OR ("Sexual and Gender Minorities"[Title/Abstract] OR "LGBT Person"[Title/Abstract] OR "Persons, LGBT"[Title/Abstract] OR "LGBTQ Person"[Title/Abstract] OR "Person, LGBTQ"[Title/Abstract] OR "Persons, LGBTQ"[Title/Abstract] OR "Non-Heterosexual Persons"[Title/Abstract] OR "Non Heterosexual Persons"[Title/Abstract] OR "LBG Persons"[Title/Abstract] OR "Sexual Minorities"[Title/Abstract] OR "Minorities, Sexual"[Title/Abstract] OR "Minority, Sexual"[Title/Abstract] OR "Sexual Minority"[Title/Abstract] OR "Non-Heterosexuals"[Title/Abstract] OR "Non Heterosexuals"[Title/Abstract] OR "Non-Heterosexual"[Title/Abstract] OR "Sexual Dissidents"[Title/Abstract] OR "Sexual Dissident"[Title/Abstract] OR "GLBT Persons"[Title/Abstract] OR "GLBT Person"[Title/Abstract] OR Gays[Title/Abstract] OR Gay[Title/Abstract] OR "Men Who Have Sex With Men"[Title/Abstract] OR Lesbians[Title/Abstract] OR Lesbian[Title/Abstract] OR "Women Who Have Sex With Women"[Title/Abstract] OR Homosexuals[Title/Abstract] OR Homosexual[Title/Abstract])) OR ((("sexual and gender minority"[Title/Abstract]) OR ("LGBTQIA+ people"[Title/Abstract] OR pansexual[Title/Abstract] OR questioning[Title/Abstract] OR " transgender AND intersex"[Title/Abstract] OR transgender[Title/Abstract] OR "GLBTI+"[Title/Abstract] OR "GLBTQ+"[Title/Abstract] OR "LGBTI+"[Title/Abstract] OR "LGBTIQ+"[Title/Abstract] OR "LGBTIQA+"[Title/Abstract] OR "LGBTIQQ"[Title/Abstract] OR "LGBTQ people"[Title/Abstract] OR "LGBTQ+"[Title/Abstract] OR "LGBTQ2"[Title/Abstract] OR "LGBTQ2S"[Title/Abstract] OR "LGBTQ2SIA+"[Title/Abstract] OR "LGBTQA"[Title/Abstract] OR "LGBTQAI"[Title/Abstract] OR "LGBTQIA"[Title/Abstract] OR "LGBTQIA+"[Title/Abstract] OR "LGBTQIA2S+"[Title/Abstract] OR "LGBTQQ"[Title/Abstract] OR "LGBTQQIA"[Title/Abstract] OR "LGBTTQ+"[Title/Abstract] OR "LGTBQA"[Title/Abstract] OR asexual[Title/Abstract] OR "two-spirit"[Title/Abstract] OR "2-spirit"[Title/Abstract] OR "2 spirit"[Title/Abstract])) OR (LGBTQIAPN+[Title/Abstract]))) OR ((Homosexuality[MeSH Terms]) OR (Homosexuality[Title/Abstract] OR "Ego-Dystonic Homosexuality"[Title/Abstract]))) OR ((Homosexuality, Female[MeSH Terms]) OR ("Homosexuality, Female"[Title/Abstract] OR "Female Homosexuality"[Title/Abstract] OR Lesbianism[Title/Abstract]))) OR ((((Homosexuality, Male[MeSH Terms]) OR ("Homosexuality, Male"[Title/Abstract] OR "Male Homosexuality"[Title/Abstract])) OR ("gender nonbinary"[Title/Abstract] OR "gender non binary"[Title/Abstract] OR "gender non conforming"[Title/Abstract] OR "gender nonconforming"[Title/Abstract] OR "non binary AFAB"[Title/Abstract] OR "non binary gender"[Title/Abstract] OR "non binary gender identity"[Title/Abstract] OR "non binary individuals"[Title/Abstract] OR "non binary people"[Title/Abstract] OR "non conforming gender"[Title/Abstract] OR "nonbinary AFAB"[Title/Abstract] OR "nonbinary gender"[Title/Abstract] OR "nonbinary individuals"[Title/Abstract] OR "nonbinary people"[Title/Abstract])) OR ("gender expansive youth"[Title/Abstract] OR "gender-expansive people"[Title/Abstract] OR "gender-fluid"[Title/Abstract] OR "gender-queer"[Title/Abstract] OR "gender-questioning"[Title/Abstract] OR "genderexpansive"[Title/Abstract] OR genderfluid[Title/Abstract] OR genderqueer[Title/Abstract] OR genderquestioning[Title/Abstract] OR "TGNB people"[Title/Abstract] OR "TNB individuals"[Title/Abstract] OR "TNB people"[Title/Abstract] OR "transgender and gender non-conforming"[Title/Abstract] OR "transgender and gender nonconforming"[Title/Abstract] OR "transgender and nonbinary"[Title/Abstract]))) OR (("Gender Identity"[MeSH Terms]) OR ("Gender Identity"[Title/Abstract]))	118.252
#3	#1 OR #2	454.381
#4	(((Vulnerable Populations[MeSH Terms]) OR ("Vulnerable Populations"[Title/Abstract] OR "Vulnerable Population"[Title/Abstract] OR "Disadvantaged Populations"[Title/Abstract] OR "Disadvantaged Population"[Title/Abstract] OR "Sensitive Populations"[Title/Abstract] OR "Sensitive Population"[Title/Abstract] OR "Sensitive Population Groups"[Title/Abstract] OR	228.105

	"Sensitive Population Group"[Title/Abstract] OR "Underserved Population"[Title/Abstract] OR "Underserved Populations"[Title/Abstract])) OR ("vulnerable minorities"[Title/Abstract] OR "vulnerable minority"[Title/Abstract] OR "vulnerable minority population"[Title/Abstract] OR "vulnerable people"[Title/Abstract] OR "vulnerable person"[Title/Abstract] OR "vulnerable persons"[Title/Abstract])) OR (((Social Vulnerability[MeSH Terms]) OR ("Social Vulnerability"[Title/Abstract] OR "Social Vulnerabilities"[Title/Abstract])) OR (Vulnerability[Title/Abstract] OR Vulnerable[Title/Abstract]))	
#5	((Health Inequities[MeSH Terms]) OR ("Health Inequities"[Title/Abstract] OR "Health Inequity"[Title/Abstract] OR "Health Inequalities"[Title/Abstract] OR "Health Inequality"[Title/Abstract] OR "Health Disparities"[Title/Abstract] OR "Health Disparity"[Title/Abstract])) OR ("disparity in health"[Title/Abstract] OR "health status disparities"[Title/Abstract] OR "health status disparity"[Title/Abstract] OR "inequality in health"[Title/Abstract] OR "inequity in health"[Title/Abstract] OR "socioeconomic disparities in health"[Title/Abstract])	79.242
#6	(health[Title/Abstract]) AND (Inequities[Title/Abstract] OR Inequity[Title/Abstract] OR Inequalities[Title/Abstract] OR Inequality[Title/Abstract] OR Disparities[Title/Abstract] OR Disparity[Title/Abstract])	116.162
#7	#**5 OR #6**	**138.626**
#8	#**4 AND #7**	12.017
#9	((((((((Teaching[MeSH Terms]) OR (Teaching[Title/Abstract] OR "Training Techniques"[Title/Abstract] OR "Training Technique"[Title/Abstract] OR "Training Technics"[Title/Abstract] OR "Training Technic"[Title/Abstract] OR Pedagogy[Title/Abstract] OR Pedagogies[Title/Abstract] OR "Teaching Methods"[Title/Abstract] OR "Teaching Method"[Title/Abstract] OR "Academic Training"[Title/Abstract] OR "Training Activities"[Title/Abstract] OR "Training Activity"[Title/Abstract] OR "Educational Techniques"[Title/Abstract] OR "Educational Technique"[Title/Abstract] OR "Educational Technics"[Title/Abstract] OR "Educational Technic"[Title/Abstract])) OR (((Curriculum[MeSH Terms]) OR (Curriculum[Title/Abstract] OR Curricula[Title/Abstract] OR "Short-Term Courses"[Title/Abstract] OR "Short Term Courses"[Title/Abstract])) OR ("competency-based education"[Title/Abstract] OR "integrated curriculum"[Title/Abstract]))) OR ((Education, Continuing[MeSH Terms]) OR ("Education, Continuing"[Title/Abstract] OR "Continuous Learning"[Title/Abstract] OR "Lifelong Learning"[Title/Abstract] OR "Life-Long Learning"[Title/Abstract] OR "Life Long Learning" "Continuing Education"[Title/Abstract]))) OR ((Education, Professional[MeSH Terms]) OR ("Education, Professional"[Title/Abstract] OR "Professional Education"[Title/Abstract]))) OR (((Interprofessional Education[MeSH Terms]) OR ("Interprofessional Education"[Title/Abstract] OR "Education, Interprofessional"[Title/Abstract])) OR ("inter-professional education"[Title/Abstract] OR "cross training"[Title/Abstract] OR multi-skilling[Title/Abstract] OR multiskilling[Title/Abstract]))) OR ("Professional Training"[Title/Abstract])) OR ((Schools[MeSH Terms]) OR (Schools[Title/Abstract] OR School[Title/Abstract] OR "Secondary School"[Title/Abstract] OR "Secondary Schools"[Title/Abstract]))) OR ((Health Personnel[MeSH Terms]) OR ("Health Personnel"[Title/Abstract] OR "Healthcare Workers"[Title/Abstract] OR "Healthcare Worker"[Title/Abstract] OR "Health Care Providers"[Title/Abstract] OR "Health Care Provider"[Title/Abstract] OR "Healthcare Providers"[Title/Abstract] OR "Healthcare Provider"[Title/Abstract] OR "Health Care Professionals"[Title/Abstract] OR "Health Care Professional"[Title/Abstract]))	1,548811
#10	#**3 AND #8 AND #9**	**372**

PUBMED PMC (NIH/NLM)

SEARCH	QUERY	RECORDS RETRIEVED
#1	(((Black People[MeSH Terms]) OR ("Black People"[Title/Abstract] OR "Black Peoples"[Title/Abstract] OR "People, Black"[Title/Abstract] OR "Black Person"[Title/Abstract] OR "Black Persons"[Title/Abstract] OR "Negroid Race"[Title/Abstract] OR "Negroid Races"[Title/Abstract] OR "Race, Negroid"[Title/Abstract] OR "African Continental Ancestry Group"[Title/Abstract])) OR ("Black Population"[Title/Abstract] OR "Black man"[Title/Abstract] OR "Black race"[Title/Abstract] OR Negroid[Title/Abstract] OR "Negroid race"[Title/Abstract] OR Negroids[Title/Abstract])) OR (((Black or African American[MeSH Terms]) OR ("Black or African American"[Title/Abstract] OR "Black Americans"[Title/Abstract] OR "American, Black"[Title/Abstract] OR "Black American"[Title/Abstract] OR Blacks[Title/Abstract] OR Negroes[Title/Abstract] OR Negro[Title/Abstract] OR "African Americans"[Title/Abstract] OR "African American"[Title/Abstract] OR "American, African"[Title/Abstract] OR "Afro-American"[Title/Abstract] OR "Afro American"[Title/Abstract] OR "Afro-Americans"[Title/Abstract] OR "Afro Americans"[Title/Abstract] OR "African-Americans"[Title/Abstract] OR "African-American"[Title/Abstract])) OR ("American blacks"[Title/Abstract] OR "American Negro"[Title/Abstract] OR "black American"[Title/Abstract] OR "black or African American"[Title/Abstract]))	2.131.265
#2	((((((Sexual and Gender Minorities[MeSH Terms]) OR ("Sexual and Gender Minorities"[Title/Abstract] OR "LGBT Person"[Title/Abstract] OR "Persons, LGBT"[Title/Abstract] OR "LGBTQ Person"[Title/Abstract] OR "Person, LGBTQ"[Title/Abstract] OR "Persons, LGBTQ"[Title/Abstract] OR "Non-Heterosexual Persons"[Title/Abstract] OR "Non Heterosexual Persons"[Title/Abstract] OR "LBG Persons"[Title/Abstract] OR "Sexual Minorities"[Title/Abstract] OR "Minorities, Sexual"[Title/Abstract] OR "Minority, Sexual"[Title/Abstract] OR "Sexual Minority"[Title/Abstract] OR "Non-Heterosexuals"[Title/Abstract] OR "Non Heterosexuals"[Title/Abstract] OR "Non-Heterosexual"[Title/Abstract] OR "Sexual Dissidents"[Title/Abstract] OR "Sexual Dissident"[Title/Abstract] OR "GLBT Persons"[Title/Abstract] OR "GLBT Person"[Title/Abstract] OR Gays[Title/Abstract] OR Gay[Title/Abstract] OR "Men Who Have Sex With Men"[Title/Abstract] OR Lesbians[Title/Abstract] OR Lesbian[Title/Abstract] OR "Women Who Have Sex With Women"[Title/Abstract] OR Homosexuals[Title/Abstract] OR Homosexual[Title/Abstract])) OR ((("sexual and gender minority"[Title/Abstract]) OR ("LGBTQIA+ people"[Title/Abstract] OR pansexual[Title/Abstract] OR questioning[Title/Abstract] OR " transgender AND intersex"[Title/Abstract] OR transgender[Title/Abstract] OR "GLBTI+"[Title/Abstract] OR "GLBTQ+"[Title/Abstract] OR "LGBTI+"[Title/Abstract] OR "LGBTIQ+"[Title/Abstract] OR "LGBTIQA+"[Title/Abstract] OR "LGBTIQQ"[Title/Abstract] OR "LGBTQ people"[Title/Abstract] OR "LGBTQ+"[Title/Abstract] OR "LGBTQ2"[Title/Abstract] OR "LGBTQ2S"[Title/Abstract] OR "LGBTQ2SIA+"[Title/Abstract] OR "LGBTQA"[Title/Abstract] OR "LGBTQAI"[Title/Abstract] OR "LGBTQIA"[Title/Abstract] OR "LGBTQIA+"[Title/Abstract] OR "LGBTQIA2S+"[Title/Abstract] OR "LGBTQQ"[Title/Abstract] OR "LGBTQQIA"[Title/Abstract] OR "LGBTTQ+"[Title/Abstract] OR "LGTBQA"[Title/Abstract] OR asexual[Title/Abstract] OR "two-spirit"[Title/Abstract] OR "2-spirit"[Title/Abstract] OR "2 spirit"[Title/Abstract])) OR (LGBTQIAPN+[Title/Abstract]))) OR ((Homosexuality[MeSH Terms]) OR (Homosexuality[Title/Abstract] OR "Ego-Dystonic Homosexuality"[Title/Abstract]))) OR ((Homosexuality, Female[MeSH Terms]) OR ("Homosexuality, Female"[Title/Abstract] OR "Female Homosexuality"[Title/Abstract] OR Lesbianism[Title/Abstract]))) OR ((((Homosexuality, Male[MeSH Terms]) OR ("Homosexuality, Male"[Title/Abstract] OR "Male Homosexuality"[Title/Abstract])) OR ("gender nonbinary"[Title/Abstract] OR "gender non binary"[Title/Abstract] OR "gender non conforming"[Title/Abstract] OR "gender nonconforming"[Title/Abstract] OR "non binary AFAB"[Title/Abstract] OR "non binary gender"[Title/Abstract] OR "non binary gender identity"[Title/Abstract] OR "non binary	24.282

	individuals"[Title/Abstract] OR "non binary people"[Title/Abstract] OR "non conforming gender"[Title/Abstract] OR "nonbinary AFAB"[Title/Abstract] OR "nonbinary gender"[Title/Abstract] OR "nonbinary individuals"[Title/Abstract] OR "nonbinary people"[Title/Abstract])) OR ("gender expansive youth"[Title/Abstract] OR "gender-expansive people"[Title/Abstract] OR "gender-fluid"[Title/Abstract] OR "gender-queer"[Title/Abstract] OR "gender-questioning"[Title/Abstract] OR "genderexpansive"[Title/Abstract] OR genderfluid[Title/Abstract] OR genderqueer[Title/Abstract] OR genderquestioning[Title/Abstract] OR "TGNB people"[Title/Abstract] OR "TNB individuals"[Title/Abstract] OR "TNB people"[Title/Abstract] OR "transgender and gender non-conforming"[Title/Abstract] OR "transgender and gender nonconforming"[Title/Abstract] OR "transgender and nonbinary"[Title/Abstract]))) OR (("Gender Identity"[MeSH Terms]) OR ("Gender Identity"[Title/Abstract]))	
#3	#1 OR #2	2.144.851
#4	(((Vulnerable Populations[MeSH Terms]) OR ("Vulnerable Populations"[Title/Abstract] OR "Vulnerable Population"[Title/Abstract] OR "Disadvantaged Populations"[Title/Abstract] OR "Disadvantaged Population"[Title/Abstract] OR "Sensitive Populations"[Title/Abstract] OR "Sensitive Population"[Title/Abstract] OR "Sensitive Population Groups"[Title/Abstract] OR "Sensitive Population Group"[Title/Abstract] OR "Underserved Population"[Title/Abstract] OR "Underserved Populations"[Title/Abstract])) OR ("vulnerable minorities"[Title/Abstract] OR "vulnerable minority"[Title/Abstract] OR "vulnerable minority population"[Title/Abstract] OR "vulnerable people"[Title/Abstract] OR "vulnerable person"[Title/Abstract] OR "vulnerable persons"[Title/Abstract])) OR (((Social Vulnerability[MeSH Terms]) OR ("Social Vulnerability"[Title/Abstract] OR "Social Vulnerabilities"[Title/Abstract])) OR (Vulnerability[Title/Abstract] OR Vulnerable[Title/Abstract]))	13.996
#5	((Health Inequities[MeSH Terms]) OR ("Health Inequities"[Title/Abstract] OR "Health Inequity"[Title/Abstract] OR "Health Inequalities"[Title/Abstract] OR "Health Inequality"[Title/Abstract] OR "Health Disparities"[Title/Abstract] OR "Health Disparity"[Title/Abstract])) OR ("disparity in health"[Title/Abstract] OR "health status disparities"[Title/Abstract] OR "health status disparity"[Title/Abstract] OR "inequality in health"[Title/Abstract] OR "inequity in health"[Title/Abstract] OR "socioeconomic disparities in health"[Title/Abstract])	21.098
#6	(health[Title/Abstract]) AND (Inequities[Title/Abstract] OR Inequity[Title/Abstract] OR Inequalities[Title/Abstract] OR Inequality[Title/Abstract] OR Disparities[Title/Abstract] OR Disparity[Title/Abstract])	8.106
#7	**#5 OR #6**	**23.582**
#8	**#4 AND #7**	**597**
#9	((((((((Teaching[MeSH Terms]) OR (Teaching[Title/Abstract] OR "Training Techniques"[Title/Abstract] OR "Training Technique"[Title/Abstract] OR "Training Technics"[Title/Abstract] OR "Training Technic"[Title/Abstract] OR Pedagogy[Title/Abstract] OR Pedagogies[Title/Abstract] OR "Teaching Methods"[Title/Abstract] OR "Teaching Method"[Title/Abstract] OR "Academic Training"[Title/Abstract] OR "Training Activities"[Title/Abstract] OR "Training Activity"[Title/Abstract] OR "Educational Techniques"[Title/Abstract] OR "Educational Technique"[Title/Abstract] OR "Educational Technics"[Title/Abstract] OR "Educational Technic"[Title/Abstract])) OR (((Curriculum[MeSH Terms]) OR (Curriculum[Title/Abstract] OR Curricula[Title/Abstract] OR "Short-Term Courses"[Title/Abstract] OR "Short Term Courses"[Title/Abstract])) OR ("competency-based education"[Title/Abstract] OR "integrated curriculum"[Title/Abstract]))) OR ((Education, Continuing[MeSH Terms]) OR ("Education, Continuing"[Title/Abstract] OR "Continuous Learning"[Title/Abstract] OR "Lifelong Learning"[Title/Abstract] OR "Life-Long Learning"[Title/Abstract] OR "Life Long Learning" "Continuing Education"[Title/Abstract]))) OR ((Education, Professional[MeSH Terms]) OR ("Education, Professional"[Title/Abstract] OR	236.002

	"Professional Education"[Title/Abstract]))) OR (((Interprofessional Education[MeSH Terms]) OR ("Interprofessional Education"[Title/Abstract] OR "Education, Interprofessional"[Title/Abstract])) OR ("inter-professional education"[Title/Abstract] OR "cross training"[Title/Abstract] OR multi-skilling[Title/Abstract] OR multiskilling[Title/Abstract]))) OR ("Professional Training"[Title/Abstract])) OR ((Schools[MeSH Terms]) OR (Schools[Title/Abstract] OR School[Title/Abstract] OR "Secondary School"[Title/Abstract] OR "Secondary Schools"[Title/Abstract]))) OR ((Health Personnel[MeSH Terms]) OR ("Health Personnel"[Title/Abstract] OR "Healthcare Workers"[Title/Abstract] OR "Healthcare Worker"[Title/Abstract] OR "Health Care Providers"[Title/Abstract] OR "Health Care Provider"[Title/Abstract] OR "Healthcare Providers"[Title/Abstract] OR "Healthcare Provider"[Title/Abstract] OR "Health Care Professionals"[Title/Abstract] OR "Health Care Professional"[Title/Abstract]))	
#10	**#3 AND #8 AND #9**	**17**

EMBASE (Elsevier)

SEARCH	QUERY	RECORDS RETRIEVED
#1	'black person'/syn OR 'black'/exp OR 'african american'/syn	241.307
#2	'sexual and gender minority'/exp OR 'lgbtqia+ people'/syn OR lgbtqiapn+:ti,ab,kw OR 'homosexuality'/syn OR 'lesbianism'/syn OR 'male homosexuality'/syn OR 'gender nonbinary'/exp OR 'gender nonbinary'/syn OR 'gender identity'/syn	107.656
#3	**#1 OR #2**	**344.222**
#4	'social vulnerability'/syn OR vulnerability:ti,ab,kw OR vulnerable:ti,ab,kw	272.147
#5	'health disparity'/syn	81.857
#6	health:ti,ab,kw AND (inequities:ti,ab,kw OR inequity:ti,ab,kw OR inequalities:ti,ab,kw OR inequality:ti,ab,kw OR disparities:ti,ab,kw OR disparity:ti,ab,kw)	141.623
#7	**#5 OR #6**	168.600
#8	**#4 AND #7**	11.140
#9	'teaching'/syn OR 'curriculum'/syn OR 'continuing education'/syn OR 'vocational education'/syn OR 'interprofessional education'/syn OR 'professional training':ti,ab,kw OR 'school'/syn OR 'health care personnel'/syn	11.196.991
#10	**#3 AND #8 AND #9**	**1.174**

BVS/VHL – Biblioteca Virtual em Saúde/Virtual Health Library

SEARCH	QUERY	RECORDS RETRIEVED
#1	("Black People" OR "Black Peoples" OR "People, Black" OR "Black Person" OR "Black Persons" OR "Negroid Race" OR "Negroid Races" OR "Race, Negroid" OR "African Continental Ancestry Group") OR ("Black Population" OR "Black man" OR "Black race" OR negroid OR "Negroid race" OR negroids) OR ("Black or African American" OR "Black Americans" OR "American, Black" OR "Black American" OR blacks OR negroes OR negro OR "African Americans" OR "African American" OR "American, African" OR "Afro-American" OR "Afro American" OR "Afro-Americans" OR "Afro Americans" OR "African-Americans" OR "African-American") OR ("American blacks" OR "American Negro" OR "black American" OR "black or African American")	277.774

#2	("Sexual and Gender Minorities" OR "LGBT Person" OR "Persons, LGBT" OR "LGBTQ Person" OR "Person, LGBTQ" OR "Persons, LGBTQ" OR "Non-Heterosexual Persons" OR "Non Heterosexual Persons" OR "LBG Persons" OR "Sexual Minorities" OR "Minorities, Sexual" OR "Minority, Sexual" OR "Sexual Minority" OR "Non-Heterosexuals" OR "Non Heterosexuals" OR "Non-Heterosexual" OR "Sexual Dissidents" OR "Sexual Dissident" OR "GLBT Persons" OR "GLBT Person" OR gays OR gay OR "Men Who Have Sex With Men" OR lesbians OR lesbian OR "Women Who Have Sex With Women" OR homosexuals OR homosexual) OR ("sexual and gender minority") OR ("LGBTQIA+ people" OR pansexual OR questioning OR " transgender and intersex" OR transgender OR "GLBTI+" OR "GLBTQ+" OR "LGBTI+" OR "LGBTIQ+" OR "LGBTIQA+" OR "LGBTIQQ" OR "LGBTQ people" OR "LGBTQ+" OR "LGBTQ2" OR "LGBTQ2S" OR "LGBTQ2SIA+" OR "LGBTQA" OR "LGBTQAI" OR "LGBTQIA" OR "LGBTQIA+" OR "LGBTQIA2S+" OR "LGBTQQ" OR "LGBTQQIA" OR "LGBTTQ+" OR "LGTBQA" OR asexual OR "two-spirit" OR "2-spirit" OR "2 spirit") OR (lgbtqiapn+) OR (homosexuality OR "Ego-Dystonic Homosexuality") OR ("Homosexuality, Female" OR "Female Homosexuality" OR lesbianism) OR ("Homosexuality, Male" OR "Male Homosexuality") OR ("gender nonbinary" OR "gender non binary" OR "gender non conforming" OR "gender nonconforming" OR "non binary AFAB" OR "non binary gender" OR "non binary gender identity" OR "non binary individuals" OR "non binary people" OR "non conforming gender" OR "nonbinary AFAB" OR "nonbinary gender" OR "nonbinary individuals" OR "nonbinary people") OR ("gender expansive youth" OR "gender-expansive people" OR "gender-fluid" OR "gender-queer" OR "gender-questioning" OR "genderexpansive" OR genderfluid OR genderqueer OR genderquestioning OR "TGNB people" OR "TNB individuals" OR "TNB people" OR "transgender and gender non-conforming" OR "transgender and gender nonconforming" OR "transgender and nonbinary") OR ("Gender Identity")	127.984
**#3**	**#1 OR #2**	**401.771**
#4	("Vulnerable Populations" OR "Vulnerable Population" OR "Disadvantaged Populations" OR "Disadvantaged Population" OR "Sensitive Populations" OR "Sensitive Population" OR "Sensitive Population Groups" OR "Sensitive Population Group" OR "Underserved Population" OR "Underserved Populations") OR ("vulnerable minorities" OR "vulnerable minority" OR "vulnerable minority population" OR "vulnerable people" OR "vulnerable person" OR "vulnerable persons") OR ("Social Vulnerability" OR "Social Vulnerabilities") OR (vulnerability OR vulnerable)	257.740
#5	("Health Inequities" OR "Health Inequity" OR "Health Inequalities" OR "Health Inequality" OR "Health Disparities" OR "Health Disparity") OR ("disparity in health" OR "health status disparities" OR "health status disparity" OR "inequality in health" OR "inequity in health" OR "socioeconomic disparities in health")	64.434
#6	(health) AND (inequities OR inequity OR inequalities OR inequality OR disparities OR disparity)	341.957
**#7**	**#5 OR #6**	**341.957**
**#8**	**#4 AND #7**	**21.508**
#9	(teaching OR "Training Techniques" OR "Training Technique" OR "Training Technics" OR "Training Technic" OR pedagogy OR pedagogies OR "Teaching Methods" OR "Teaching Method" OR "Academic Training" OR "Training Activities" OR "Training Activity" OR "Educational Techniques" OR "Educational Technique" OR "Educational Technics" OR "Educational Technic") OR (curriculum OR curricula OR "Short-Term Courses" OR "Short Term Courses") OR ("competency-based education" OR "integrated curriculum") OR ("Education, Continuing" OR "Continuous Learning" OR "Lifelong Learning" OR "Life-Long Learning" OR "Life Long Learning" "Continuing Education") OR ("Education, Professional" OR "Professional Education") OR ("Interprofessional Education" OR "Education, Interprofessional") OR ("inter-professional education" OR "cross training" OR multi-skilling OR multiskilling) OR ("Professional Training") OR (schools OR school OR "Secondary School" OR "Secondary Schools") OR ("Health Personnel" OR "Healthcare Workers" OR "Healthcare Worker" OR "Health Care Providers" OR "Health Care Provider" OR "Healthcare Providers" OR "Healthcare Provider" OR "Health Care Professionals" OR "Health Care Professional")	1.497.506
**#10**	**#3 AND #7 AND #9**	**427**

CINAHL (EBSCOhost)

SEARCH	QUERY	RECORDS RETRIEVED
#1	XB (("Black People" OR "Black Peoples" OR "People, Black" OR "Black Person" OR "Black Persons" OR "Negroid Race" OR "Negroid Races" OR "Race, Negroid" OR "African Continental Ancestry Group") OR ("Black Population" OR "Black man" OR "Black race" OR negroid OR "Negroid race" OR negroids) OR ("Black or African American" OR "Black Americans" OR "American, Black" OR "Black American" OR blacks OR negroes OR negro OR "African Americans" OR "African American" OR "American, African" OR "Afro-American" OR "Afro American" OR "Afro-Americans" OR "Afro Americans" OR "African-Americans" OR "African-American") OR ("American blacks" OR "American Negro" OR "black American" OR "black or African American")) OR SU (("Black People" OR "Black Peoples" OR "People, Black" OR "Black Person" OR "Black Persons" OR "Negroid Race" OR "Negroid Races" OR "Race, Negroid" OR "African Continental Ancestry Group") OR ("Black Population" OR "Black man" OR "Black race" OR negroid OR "Negroid race" OR negroids) OR ("Black or African American" OR "Black Americans" OR "American, Black" OR "Black American" OR blacks OR negroes OR negro OR "African Americans" OR "African American" OR "American, African" OR "Afro-American" OR "Afro American" OR "Afro-Americans" OR "Afro Americans" OR "African-Americans" OR "African-American") OR ("American blacks" OR "American Negro" OR "black American" OR "black or African American"))	87,873
#2	XB (("Sexual and Gender Minorities" OR "LGBT Person" OR "Persons, LGBT" OR "LGBTQ Person" OR "Person, LGBTQ" OR "Persons, LGBTQ" OR "Non-Heterosexual Persons" OR "Non Heterosexual Persons" OR "LBG Persons" OR "Sexual Minorities" OR "Minorities, Sexual" OR "Minority, Sexual" OR "Sexual Minority" OR "Non-Heterosexuals" OR "Non Heterosexuals" OR "Non-Heterosexual" OR "Sexual Dissidents" OR "Sexual Dissident" OR "GLBT Persons" OR "GLBT Person" OR gays OR gay OR "Men Who Have Sex With Men" OR lesbians OR lesbian OR "Women Who Have Sex With Women" OR homosexuals OR homosexual) OR ("sexual and gender minority") OR ("LGBTQIA+ people" OR pansexual OR questioning OR " transgender and intersex" OR transgender OR "GLBTI+" OR "GLBTQ+" OR "LGBTI+" OR "LGBTIQ+" OR "LGBTIQA+" OR "LGBTIQQ" OR "LGBTQ people" OR "LGBTQ+" OR "LGBTQ2" OR "LGBTQ2S" OR "LGBTQ2SIA+" OR "LGBTQA" OR "LGBTQAI" OR "LGBTQIA" OR "LGBTQIA+" OR "LGBTQIA2S+" OR "LGBTQQ" OR "LGBTQQIA" OR "LGBTTQ+" OR "LGTBQA" OR asexual OR "two-spirit" OR "2-spirit" OR "2 spirit") OR (lgbtqiapn+) OR (homosexuality OR "Ego-Dystonic Homosexuality") OR ("Homosexuality, Female" OR "Female Homosexuality" OR lesbianism) OR ("Homosexuality, Male" OR "Male Homosexuality") OR ("gender nonbinary" OR "gender non binary" OR "gender non conforming" OR "gender nonconforming" OR "non binary AFAB" OR "non binary gender" OR "non binary gender identity" OR "non binary individuals" OR "non binary people" OR "non conforming gender" OR "nonbinary AFAB" OR "nonbinary gender" OR "nonbinary individuals" OR "nonbinary people") OR ("gender expansive youth" OR "gender-expansive people" OR "gender-fluid" OR "gender-queer" OR "gender-questioning" OR "genderexpansive" OR genderfluid OR genderqueer OR genderquestioning OR "TGNB people" OR "TNB individuals" OR "TNB people" OR "transgender and gender non-conforming" OR "transgender and gender nonconforming" OR "transgender and nonbinary") OR ("Gender Identity")) OR SU (("Sexual and Gender Minorities" OR "LGBT Person" OR "Persons, LGBT" OR "LGBTQ Person" OR "Person, LGBTQ" OR "Persons, LGBTQ" OR "Non-Heterosexual Persons" OR "Non Heterosexual Persons" OR "LBG Persons" OR "Sexual Minorities" OR "Minorities, Sexual" OR "Minority, Sexual" OR	42,503

	"Sexual Minority" OR "Non-Heterosexuals" OR "Non Heterosexuals" OR "Non-Heterosexual" OR "Sexual Dissidents" OR "Sexual Dissident" OR "GLBT Persons" OR "GLBT Person" OR gays OR gay OR "Men Who Have Sex With Men" OR lesbians OR lesbian OR "Women Who Have Sex With Women" OR homosexuals OR homosexual) OR ("sexual and gender minority") OR ("LGBTQIA+ people" OR pansexual OR questioning OR " transgender and intersex" OR transgender OR "GLBTI+" OR "GLBTQ+" OR "LGBTI+" OR "LGBTIQ+" OR "LGBTIQA+" OR "LGBTIQQ" OR "LGBTQ people" OR "LGBTQ+" OR "LGBTQ2" OR "LGBTQ2S" OR "LGBTQ2SIA+" OR "LGBTQA" OR "LGBTQAI" OR "LGBTQIA" OR "LGBTQIA+" OR "LGBTQIA2S+" OR "LGBTQQ" OR "LGBTQQIA" OR "LGBTTQ+" OR "LGTBQA" OR asexual OR "two-spirit" OR "2-spirit" OR "2 spirit") OR (lgbtqiapn+) OR (homosexuality OR "Ego-Dystonic Homosexuality") OR ("Homosexuality, Female" OR "Female Homosexuality" OR lesbianism) OR ("Homosexuality, Male" OR "Male Homosexuality") OR ("gender nonbinary" OR "gender non binary" OR "gender non conforming" OR "gender nonconforming" OR "non binary AFAB" OR "non binary gender" OR "non binary gender identity" OR "non binary individuals" OR "non binary people" OR "non conforming gender" OR "nonbinary AFAB" OR "nonbinary gender" OR "nonbinary individuals" OR "nonbinary people") OR ("gender expansive youth" OR "gender-expansive people" OR "gender-fluid" OR "gender-queer" OR "gender-questioning" OR "genderexpansive" OR genderfluid OR genderqueer OR genderquestioning OR "TGNB people" OR "TNB individuals" OR "TNB people" OR "transgender and gender non-conforming" OR "transgender and gender nonconforming" OR "transgender and nonbinary") OR ("Gender Identity"))	
#3	**#1 OR #2**	**127,695**
#4	XB (("Vulnerable Populations" OR "Vulnerable Population" OR "Disadvantaged Populations" OR "Disadvantaged Population" OR "Sensitive Populations" OR "Sensitive Population" OR "Sensitive Population Groups" OR "Sensitive Population Group" OR "Underserved Population" OR "Underserved Populations") OR ("vulnerable minorities" OR "vulnerable minority" OR "vulnerable minority population" OR "vulnerable people" OR "vulnerable person" OR "vulnerable persons") OR ("Social Vulnerability" OR "Social Vulnerabilities") OR (vulnerability OR vulnerable)) OR SU (("Vulnerable Populations" OR "Vulnerable Population" OR "Disadvantaged Populations" OR "Disadvantaged Population" OR "Sensitive Populations" OR "Sensitive Population" OR "Sensitive Population Groups" OR "Sensitive Population Group" OR "Underserved Population" OR "Underserved Populations") OR ("vulnerable minorities" OR "vulnerable minority" OR "vulnerable minority population" OR "vulnerable people" OR "vulnerable person" OR "vulnerable persons") OR ("Social Vulnerability" OR "Social Vulnerabilities") OR (vulnerability OR vulnerable))	71,714
#5	XB (("Health Inequities" OR "Health Inequity" OR "Health Inequalities" OR "Health Inequality" OR "Health Disparities" OR "Health Disparity") OR ("disparity in health" OR "health status disparities" OR "health status disparity" OR "inequality in health" OR "inequity in health" OR "socioeconomic disparities in health")) OR SU (("Health Inequities" OR "Health Inequity" OR "Health Inequalities" OR "Health Inequality" OR "Health Disparities" OR "Health Disparity") OR ("disparity in health" OR "health status disparities" OR "health status disparity" OR "inequality in health" OR "inequity in health" OR "socioeconomic disparities in health"))	19,807
#6	XB ((health) AND (inequities OR inequity OR inequalities OR inequality OR disparities OR disparity)) OR SU ((health) AND (inequities OR inequity OR inequalities OR inequality OR disparities OR disparity))	48,713
#7	**#5 OR #6**	**49,281**
#8	**#4 AND #7**	**4,032**
#9	XB ((teaching OR "Training Techniques" OR "Training Technique" OR "Training Technics" OR "Training Technic" OR pedagogy OR pedagogies OR "Teaching Methods" OR "Teaching Method" OR "Academic Training" OR "Training Activities" OR "Training Activity" OR "Educational Techniques" OR "Educational Technique" OR "Educational Technics" OR "Educational Technic") OR (curriculum OR curricula OR "Short-Term Courses" OR "Short Term Courses") OR ("competency-based education" OR "integrated curriculum") OR ("Education, Continuing" OR	647.926

	"Continuous Learning" OR "Lifelong Learning" OR "Life-Long Learning" OR "Life Long Learning" "Continuing Education") OR ("Education, Professional" OR "Professional Education") OR ("Interprofessional Education" OR "Education, Interprofessional") OR ("inter-professional education" OR "cross training" OR multi-skilling OR multiskilling) OR ("Professional Training") OR (schools OR school OR "Secondary School" OR "Secondary Schools") OR ("Health Personnel" OR "Healthcare Workers" OR "Healthcare Worker" OR "Health Care Providers" OR "Health Care Provider" OR "Healthcare Providers" OR "Healthcare Provider" OR "Health Care Professionals" OR "Health Care Professional")) OR SU ((teaching OR "Training Techniques" OR "Training Technique" OR "Training Technics" OR "Training Technic" OR pedagogy OR pedagogies OR "Teaching Methods" OR "Teaching Method" OR "Academic Training" OR "Training Activities" OR "Training Activity" OR "Educational Techniques" OR "Educational Technique" OR "Educational Technics" OR "Educational Technic") OR (curriculum OR curricula OR "Short-Term Courses" OR "Short Term Courses") OR ("competency-based education" OR "integrated curriculum") OR ("Education, Continuing" OR "Continuous Learning" OR "Lifelong Learning" OR "Life-Long Learning" OR "Life Long Learning" "Continuing Education") OR ("Education, Professional" OR "Professional Education") OR ("Interprofessional Education" OR "Education, Interprofessional") OR ("inter-professional education" OR "cross training" OR multi-skilling OR multiskilling) OR ("Professional Training") OR (schools OR school OR "Secondary School" OR "Secondary Schools") OR ("Health Personnel" OR "Healthcare Workers" OR "Healthcare Worker" OR "Health Care Providers" OR "Health Care Provider" OR "Healthcare Providers" OR "Healthcare Provider" OR "Health Care Professionals" OR "Health Care Professional"))	
#10	**#3 AND #8 AND #9**	**184**

ERIC (EBSCOhost)

SEARCH	QUERY	RECORDS RETRIEVED
#1	TI ("Black People" OR "Black Peoples" OR "People, Black" OR "Black Person" OR "Black Persons" OR "Negroid Race" OR "Negroid Races" OR "Race, Negroid" OR "African Continental Ancestry Group" OR "Black Population" OR "Black man" OR "Black race" OR Negroid OR "Negroid race" OR Negroids OR "Black or African American" OR "Black Americans" OR "American, Black" OR "Black American" OR Blacks OR Negroes OR Negro OR "African Americans" OR "African American" OR "American, African" OR "Afro-American" OR "Afro American" OR "Afro-Americans" OR "Afro Americans" OR "African-Americans" OR "African-American" OR "American blacks" OR "American Negro" OR "black American" OR "black or African American") OR SU ("Black People" OR "Black Peoples" OR "People, Black" OR "Black Person" OR "Black Persons" OR "Negroid Race" OR "Negroid Races" OR "Race, Negroid" OR "African Continental Ancestry Group" OR "Black Population" OR "Black man" OR "Black race" OR Negroid OR "Negroid race" OR Negroids OR "Black or African American" OR "Black Americans" OR "American, Black" OR "Black American" OR Blacks OR Negroes OR Negro OR "African Americans" OR "African American" OR "American, African" OR "Afro-American" OR "Afro American" OR "Afro-Americans" OR "Afro Americans" OR "African-Americans" OR "African-American" OR "American blacks" OR "American Negro" OR "black American" OR "black or African American") OR AB ("Black People" OR "Black Peoples" OR "People, Black" OR "Black Person" OR "Black Persons" OR "Negroid Race" OR "Negroid Races" OR "Race, Negroid" OR "African Continental Ancestry Group" OR "Black Population" OR "Black man" OR "Black race" OR Negroid OR "Negroid race" OR Negroids OR "Black or African American" OR "Black Americans" OR "American, Black" OR "Black American" OR Blacks OR Negroes OR Negro OR "African Americans" OR "African American" OR "American, African" OR "Afro-American" OR "Afro American" OR "Afro-Americans" OR "Afro Americans" OR "African-Americans" OR "African-American" OR "American blacks" OR "American Negro" OR "black American" OR "black or African American")	69.852

#2	TI ("Sexual and Gender Minorities" OR "LGBT Person" OR "Persons, LGBT" OR "LGBTQ Person" OR "Person, LGBTQ" OR "Persons, LGBTQ" OR "Non-Heterosexual Persons" OR "Non Heterosexual Persons" OR "LBG Persons" OR "Sexual Minorities" OR "Minorities, Sexual" OR "Minority, Sexual" OR "Sexual Minority" OR "Non-Heterosexuals" OR "Non Heterosexuals" OR "Non-Heterosexual" OR "Sexual Dissidents" OR "Sexual Dissident" OR "GLBT Persons" OR "GLBT Person" OR Gays OR Gay OR "Men Who Have Sex With Men" OR Lesbians OR Lesbian OR "Women Who Have Sex With Women" OR Homosexuals OR Homosexual OR "sexual and gender minority" OR "LGBTQIA+ people" OR pansexual OR questioning OR " transgender and intersex" OR transgender OR "GLBTI+" OR "GLBTQ+" OR "LGBTI+" OR "LGBTIQ+" OR "LGBTIQA+" OR "LGBTIQQ" OR "LGBTQ people" OR "LGBTQ+" OR "LGBTQ2" OR "LGBTQ2S" OR "LGBTQ2SIA+" OR "LGBTQA" OR "LGBTQAI" OR "LGBTQIA" OR "LGBTQIA+" OR "LGBTQIA2S+" OR "LGBTQQ" OR "LGBTQQIA" OR "LGBTTQ+" OR "LGTBQA" OR asexual OR "two-spirit" OR "2-spirit" OR "2 spirit" OR LGBTQIAPN+ OR Homosexuality OR "Ego-Dystonic Homosexuality" OR "Homosexuality, Female" OR "Female Homosexuality" OR Lesbianism OR "Homosexuality, Male" OR "Male Homosexuality" OR "gender nonbinary" OR "gender non binary" OR "gender non conforming" OR "gender nonconforming" OR "non binary AFAB" OR "non binary gender" OR "non binary gender identity" OR "non binary individuals" OR "non binary people" OR "non conforming gender" OR "nonbinary AFAB" OR "nonbinary gender" OR "nonbinary individuals" OR "nonbinary people" OR "gender expansive youth" OR "gender-expansive people" OR "gender-fluid" OR "gender-queer" OR "gender-questioning" OR "genderexpansive" OR genderfluid OR genderqueer OR genderquestioning OR "TGNB people" OR "TNB individuals" OR "TNB people" OR "transgender and gender non-conforming" OR "transgender and gender nonconforming" OR "transgender and nonbinary" OR "Gender Identity") OR SU ("Sexual and Gender Minorities" OR "LGBT Person" OR "Persons, LGBT" OR "LGBTQ Person" OR "Person, LGBTQ" OR "Persons, LGBTQ" OR "Non-Heterosexual Persons" OR "Non Heterosexual Persons" OR "LBG Persons" OR "Sexual Minorities" OR "Minorities, Sexual" OR "Minority, Sexual" OR "Sexual Minority" OR "Non-Heterosexuals" OR "Non Heterosexuals" OR "Non-Heterosexual" OR "Sexual Dissidents" OR "Sexual Dissident" OR "GLBT Persons" OR "GLBT Person" OR Gays OR Gay OR "Men Who Have Sex With Men" OR Lesbians OR Lesbian OR "Women Who Have Sex With Women" OR Homosexuals OR Homosexual OR "sexual and gender minority" OR "LGBTQIA+ people" OR pansexual OR questioning OR " transgender and intersex" OR transgender OR "GLBTI+" OR "GLBTQ+" OR "LGBTI+" OR "LGBTIQ+" OR "LGBTIQA+" OR "LGBTIQQ" OR "LGBTQ people" OR "LGBTQ+" OR "LGBTQ2" OR "LGBTQ2S" OR "LGBTQ2SIA+" OR "LGBTQA" OR "LGBTQAI" OR "LGBTQIA" OR "LGBTQIA+" OR "LGBTQIA2S+" OR "LGBTQQ" OR "LGBTQQIA" OR "LGBTTQ+" OR "LGTBQA" OR asexual OR "two-spirit" OR "2-spirit" OR "2 spirit" OR LGBTQIAPN+ OR Homosexuality OR "Ego-Dystonic Homosexuality" OR "Homosexuality, Female" OR "Female Homosexuality" OR Lesbianism OR "Homosexuality, Male" OR "Male Homosexuality" OR "gender nonbinary" OR "gender non binary" OR "gender non conforming" OR "gender nonconforming" OR "non binary AFAB" OR "non binary gender" OR "non binary gender identity" OR "non binary individuals" OR "non binary people" OR "non conforming gender" OR "nonbinary AFAB" OR "nonbinary gender" OR "nonbinary individuals" OR "nonbinary people" OR "gender expansive youth" OR "gender-expansive people" OR "gender-fluid" OR "gender-queer" OR "gender-questioning" OR "genderexpansive" OR genderfluid OR genderqueer OR genderquestioning OR "TGNB people" OR "TNB individuals" OR "TNB people" OR "transgender and gender non-conforming" OR "transgender and gender nonconforming" OR "transgender and nonbinary" OR "Gender Identity") OR AB ("Sexual and Gender Minorities" OR "LGBT Person" OR "Persons, LGBT" OR "LGBTQ Person" OR "Person, LGBTQ" OR "Persons, LGBTQ" OR "Non-Heterosexual Persons" OR "Non Heterosexual Persons" OR "LBG Persons" OR "Sexual Minorities" OR "Minorities, Sexual" OR "Minority, Sexual" OR "Sexual Minority" OR "Non-Heterosexuals" OR "Non Heterosexuals" OR "Non-Heterosexual" OR "Sexual Dissidents" OR "Sexual Dissident" OR "GLBT Persons" OR "GLBT Person" OR Gays OR Gay OR "Men Who Have Sex With Men" OR	22.769

	Lesbians OR Lesbian OR "Women Who Have Sex With Women" OR Homosexuals OR Homosexual OR "sexual and gender minority" OR "LGBTQIA+ people" OR pansexual OR questioning OR " transgender and intersex" OR transgender OR "GLBTI+" OR "GLBTQ+" OR "LGBTI+" OR "LGBTIQ+" OR "LGBTIQA+" OR "LGBTIQQ" OR "LGBTQ people" OR "LGBTQ+" OR "LGBTQ2" OR "LGBTQ2S" OR "LGBTQ2SIA+" OR "LGBTQA" OR "LGBTQAI" OR "LGBTQIA" OR "LGBTQIA+" OR "LGBTQIA2S+" OR "LGBTQQ" OR "LGBTQQIA" OR "LGBTTQ+" OR "LGTBQA" OR asexual OR "two-spirit" OR "2-spirit" OR "2 spirit" OR LGBTQIAPN+ OR Homosexuality OR "Ego-Dystonic Homosexuality" OR "Homosexuality, Female" OR "Female Homosexuality" OR Lesbianism OR "Homosexuality, Male" OR "Male Homosexuality" OR "gender nonbinary" OR "gender non binary" OR "gender non conforming" OR "gender nonconforming" OR "non binary AFAB" OR "non binary gender" OR "non binary gender identity" OR "non binary individuals" OR "non binary people" OR "non conforming gender" OR "nonbinary AFAB" OR "nonbinary gender" OR "nonbinary individuals" OR "nonbinary people" OR "gender expansive youth" OR "gender-expansive people" OR "gender-fluid" OR "gender-queer" OR "gender-questioning" OR "genderexpansive" OR genderfluid OR genderqueer OR genderquestioning OR "TGNB people" OR "TNB individuals" OR "TNB people" OR "transgender and gender non-conforming" OR "transgender and gender nonconforming" OR "transgender and nonbinary" OR "Gender Identity")	
**#3**	**#1 OR #2**	**91.645**
#4	TI ("Vulnerable Populations" OR "Vulnerable Population" OR "Disadvantaged Populations" OR "Disadvantaged Population" OR "Sensitive Populations" OR "Sensitive Population" OR "Sensitive Population Groups" OR "Sensitive Population Group" OR "Underserved Population" OR "Underserved Populations" OR "vulnerable minorities" OR "vulnerable minority" OR "vulnerable minority population" OR "vulnerable people" OR "vulnerable person" OR "vulnerable persons" OR "Social Vulnerability" OR "Social Vulnerabilities" OR Vulnerability OR Vulnerable) OR SU ("Vulnerable Populations" OR "Vulnerable Population" OR "Disadvantaged Populations" OR "Disadvantaged Population" OR "Sensitive Populations" OR "Sensitive Population" OR "Sensitive Population Groups" OR "Sensitive Population Group" OR "Underserved Population" OR "Underserved Populations" OR "vulnerable minorities" OR "vulnerable minority" OR "vulnerable minority population" OR "vulnerable people" OR "vulnerable person" OR "vulnerable persons" OR "Social Vulnerability" OR "Social Vulnerabilities" OR Vulnerability OR Vulnerable) OR AB ("Vulnerable Populations" OR "Vulnerable Population" OR "Disadvantaged Populations" OR "Disadvantaged Population" OR "Sensitive Populations" OR "Sensitive Population" OR "Sensitive Population Groups" OR "Sensitive Population Group" OR "Underserved Population" OR "Underserved Populations" OR "vulnerable minorities" OR "vulnerable minority" OR "vulnerable minority population" OR "vulnerable people" OR "vulnerable person" OR "vulnerable persons" OR "Social Vulnerability" OR "Social Vulnerabilities" OR Vulnerability OR Vulnerable)	11.000
#5	TI ("Health Inequities" OR "Health Inequity" OR "Health Inequalities" OR "Health Inequality" OR "Health Disparities" OR "Health Disparity" OR "disparity in health" OR "health status disparities" OR "health status disparity" OR "inequality in health" OR "inequity in health" OR "socioeconomic disparities in health") OR SU ("Health Inequities" OR "Health Inequity" OR "Health Inequalities" OR "Health Inequality" OR "Health Disparities" OR "Health Disparity" OR "disparity in health" OR "health status disparities" OR "health status disparity" OR "inequality in health" OR "inequity in health" OR "socioeconomic disparities in health") OR AB ("Health Inequities" OR "Health Inequity" OR "Health Inequalities" OR "Health Inequality" OR "Health Disparities" OR "Health Disparity" OR "disparity in health" OR "health status disparities" OR "health status disparity" OR "inequality in health" OR "inequity in health" OR "socioeconomic disparities in health")	906
#6	(TI health OR SU health OR AB health) AND (TI (Inequities OR Inequity OR Inequalities OR Inequality OR Disparities OR Disparity) OR SU (Inequities OR Inequity OR Inequalities OR Inequality OR Disparities OR Disparity) OR AB (Inequities OR Inequity OR Inequalities OR Inequality OR Disparities OR Disparity)	3.408
#7	**#5 OR #6**	**3**.408

#8	**#4 AND #7**	**192**
#9	TI (Teaching OR "Training Techniques" OR "Training Technique" OR "Training Technics" OR "Training Technic" OR Pedagogy OR Pedagogies OR "Teaching Methods" OR "Teaching Method" OR "Academic Training" OR "Training Activities" OR "Training Activity" OR "Educational Techniques" OR "Educational Technique" OR "Educational Technics" OR "Educational Technic" OR Curriculum OR Curricula OR "Short-Term Courses" OR "Short Term Courses" OR "competency-based education" OR "integrated curriculum" OR "Education, Continuing" OR "Continuous Learning" OR "Lifelong Learning" OR "Life-Long Learning" OR "Life Long Learning" "Continuing Education" OR "Education, Professional" OR "Professional Education" OR "Interprofessional Education" OR "Education, Interprofessional" OR "inter-professional education" OR "cross training" OR multi-skilling OR multiskilling OR "Professional Training" OR Schools OR School OR "Secondary School" OR "Secondary Schools" OR "Health Personnel" OR "Healthcare Workers" OR "Healthcare Worker" OR "Health Care Providers" OR "Health Care Provider" OR "Healthcare Providers" OR "Healthcare Provider" OR "Health Care Professionals" OR "Health Care Professional") OR SU (Teaching OR "Training Techniques" OR "Training Technique" OR "Training Technics" OR "Training Technic" OR Pedagogy OR Pedagogies OR "Teaching Methods" OR "Teaching Method" OR "Academic Training" OR "Training Activities" OR "Training Activity" OR "Educational Techniques" OR "Educational Technique" OR "Educational Technics" OR "Educational Technic" OR Curriculum OR Curricula OR "Short-Term Courses" OR "Short Term Courses" OR "competency-based education" OR "integrated curriculum" OR "Education, Continuing" OR "Continuous Learning" OR "Lifelong Learning" OR "Life-Long Learning" OR "Life Long Learning" "Continuing Education" OR "Education, Professional" OR "Professional Education" OR "Interprofessional Education" OR "Education, Interprofessional" OR "inter-professional education" OR "cross training" OR multi-skilling OR multiskilling OR "Professional Training" OR Schools OR School OR "Secondary School" OR "Secondary Schools" OR "Health Personnel" OR "Healthcare Workers" OR "Healthcare Worker" OR "Health Care Providers" OR "Health Care Provider" OR "Healthcare Providers" OR "Healthcare Provider" OR "Health Care Professionals" OR "Health Care Professional") OR AB (Teaching OR "Training Techniques" OR "Training Technique" OR "Training Technics" OR "Training Technic" OR Pedagogy OR Pedagogies OR "Teaching Methods" OR "Teaching Method" OR "Academic Training" OR "Training Activities" OR "Training Activity" OR "Educational Techniques" OR "Educational Technique" OR "Educational Technics" OR "Educational Technic" OR Curriculum OR Curricula OR "Short-Term Courses" OR "Short Term Courses" OR "competency-based education" OR "integrated curriculum" OR "Education, Continuing" OR "Continuous Learning" OR "Lifelong Learning" OR "Life-Long Learning" OR "Life Long Learning" "Continuing Education" OR "Education, Professional" OR "Professional Education" OR "Interprofessional Education" OR "Education, Interprofessional" OR "inter-professional education" OR "cross training" OR multi-skilling OR multiskilling OR "Professional Training" OR Schools OR School OR "Secondary School" OR "Secondary Schools" OR "Health Personnel" OR "Healthcare Workers" OR "Healthcare Worker" OR "Health Care Providers" OR "Health Care Provider" OR "Healthcare Providers" OR "Healthcare Provider" OR "Health Care Professionals" OR "Health Care Professional")	1.170.457
#10	**#3 AND #8 AND #9**	**20**

COCHRANE (Wiley)

SEARCH	QUERY	RECORDS RETRIEVED
#1	MeSH descriptor: [Black People] explode all trees OR MeSH descriptor: [Black or African American] explode all trees OR ("Black People" OR "Black Peoples" OR "People, Black" OR "Black Person" OR "Black Persons" OR "Negroid Race" OR "Negroid Races" OR "Race, Negroid" OR "African Continental Ancestry Group" OR "Black Population" OR "Black man" OR "Black race" OR	12.314

	Negroid OR "Negroid race" OR Negroids OR "Black or African American" OR "Black Americans" OR "American, Black" OR "Black American" OR Blacks OR Negroes OR Negro OR "African Americans" OR "African American" OR "American, African" OR "Afro-American" OR "Afro American" OR "Afro-Americans" OR "Afro Americans" OR "African-Americans" OR "African-American" OR "American blacks" OR "American Negro" OR "black American" OR "black or African American"):ti,ab,kw	
#2	MeSH descriptor: [Sexual and Gender Minorities] explode all trees OR ("Sexual and Gender Minorities" OR "LGBT Person" OR "Persons, LGBT" OR "LGBTQ Person" OR "Person, LGBTQ" OR "Persons, LGBTQ" OR "Non-Heterosexual Persons" OR "Non Heterosexual Persons" OR "LBG Persons" OR "Sexual Minorities" OR "Minorities, Sexual" OR "Minority, Sexual" OR "Sexual Minority" OR "Non-Heterosexuals" OR "Non Heterosexuals" OR "Non-Heterosexual" OR "Sexual Dissidents" OR "Sexual Dissident" OR "GLBT Persons" OR "GLBT Person" OR Gays OR Gay OR "Men Who Have Sex With Men" OR Lesbians OR Lesbian OR "Women Who Have Sex With Women" OR Homosexuals OR Homosexual):ti,ab,kw OR ("sexual and gender minority"):ti,ab,kw OR ("LGBTQIA+ people" OR pansexual OR questioning OR " transgender and intersex" OR transgender OR "GLBTI+" OR "GLBTQ+" OR "LGBTI+" OR "LGBTIQ+" OR "LGBTIQA+" OR "LGBTIQQ" OR "LGBTQ people" OR "LGBTQ+" OR "LGBTQ2" OR "LGBTQ2S" OR "LGBTQ2SIA+" OR "LGBTQA" OR "LGBTQAI" OR "LGBTQIA" OR "LGBTQIA+" OR "LGBTQIA2S+" OR "LGBTQQ" OR "LGBTQQIA" OR "LGBTTQ+" OR "LGTBQA" OR asexual OR "two-spirit" OR "2-spirit" OR "2 spirit"):ti,ab,kw OR (LGBTQIAPN):ti,ab,kw OR MeSH descriptor: [Homosexuality] in all MeSH products OR (Homosexuality OR "Ego-Dystonic Homosexuality"):ti,ab,kw OR MeSH descriptor: [Homosexuality, Female] explode all trees OR ("Homosexuality, Female" OR "Female Homosexuality" OR Lesbianism):ti,ab,kw OR MeSH descriptor: [Homosexuality, Male] explode all trees OR ("Homosexuality, Male" OR "Male Homosexuality"):ti,ab,kw OR ("gender nonbinary" OR "gender non binary" OR "gender non conforming" OR "gender nonconforming" OR "non binary AFAB" OR "non binary gender" OR "non binary gender identity" OR "non binary individuals" OR "non binary people" OR "non conforming gender" OR "nonbinary AFAB" OR "nonbinary gender" OR "nonbinary individuals" OR "nonbinary people"):ti,ab,kw OR ("gender expansive youth" OR "gender-expansive people" OR "gender-fluid" OR "gender-queer" OR "gender-questioning" OR "genderexpansive" OR genderfluid OR genderqueer OR genderquestioning OR "TGNB people" OR "TNB individuals" OR "TNB people" OR "transgender and gender non-conforming" OR "transgender and gender nonconforming" OR "transgender and nonbinary"):ti,ab,kw OR MeSH descriptor: [Gender Identity] explode all trees OR ("gender identity"):ti,ab,kw	5.321
**#3**	**#1 OR #2**	**17.352**
#4	MeSH descriptor: [Vulnerable Populations] explode all trees OR MeSH descriptor: [Social Vulnerability] explode all trees OR ("Vulnerable Populations" OR "Vulnerable Population" OR "Disadvantaged Populations" OR "Disadvantaged Population" OR "Sensitive Populations" OR "Sensitive Population" OR "Sensitive Population Groups" OR "Sensitive Population Group" OR "Underserved Population" OR "Underserved Populations" OR "vulnerable minorities" OR "vulnerable minority" OR "vulnerable minority population" OR "vulnerable people" OR "vulnerable person" OR "vulnerable persons" OR "Social Vulnerability" OR "Social Vulnerabilities" OR Vulnerability OR Vulnerable):ti,ab,kw	11.069
#5	MeSH descriptor: [Health Inequities] explode all trees OR ("Health Inequities" OR "Health Inequity" OR "Health Inequalities" OR "Health Inequality" OR "Health Disparities" OR "Health Disparity" OR "disparity in health" OR "health status disparities" OR "health status disparity" OR "inequality in health" OR "inequity in health" OR "socioeconomic disparities in health"):ti,ab,kw	2.196
#6	(health):ti,ab,kw AND (Inequities OR Inequity OR Inequalities OR Inequality OR Disparities OR Disparity):ti,ab,kw	4.949
#7	**#5 OR #6**	**4.949**
#8	**#4 AND #7**	**592**

#9	MeSH descriptor: [Teaching] explode all trees OR MeSH descriptor: [Curriculum] explode all trees OR MeSH descriptor: [Education, Continuing] explode all trees OR MeSH descriptor: [Education, Professional] in all MeSH products OR MeSH descriptor: [Interprofessional Education] explode all trees OR MeSH descriptor: [Schools] explode all trees OR MeSH descriptor: [Health Personnel] explode all trees OR (Teaching OR "Training Techniques" OR "Training Technique" OR "Training Technics" OR "Training Technic" OR Pedagogy OR Pedagogies OR "Teaching Methods" OR "Teaching Method" OR "Academic Training" OR "Training Activities" OR "Training Activity" OR "Educational Techniques" OR "Educational Technique" OR "Educational Technics" OR "Educational Technic" OR Curriculum OR Curricula OR "Short-Term Courses" OR "Short Term Courses" OR "competency-based education" OR "integrated curriculum" OR "Education, Continuing" OR "Continuous Learning" OR "Lifelong Learning" OR "Life-Long Learning" OR "Life Long Learning" "Continuing Education" OR "Education, Professional" OR "Professional Education" OR "Interprofessional Education" OR "Education, Interprofessional" OR "inter-professional education" OR "cross training" OR multi-skilling OR multiskilling OR "Professional Training" OR Schools OR School OR "Secondary School" OR "Secondary Schools" OR "Health Personnel" OR "Healthcare Workers" OR "Healthcare Worker" OR "Health Care Providers" OR "Health Care Provider" OR "Healthcare Providers" OR "Healthcare Provider" OR "Health Care Professionals" OR "Health Care Professional"):ti,ab,kw	102.013
#10	**#3 AND #8 AND #9**	**24**

Biblioteca Digital Brasileira de Teses e Dissertações – BDBTD (IBICT)

SEARCH	QUERY	RECORDS RETRIEVE D
#1	(Todos os campos: "População Negra" OR Negro OR Afro-Americano" OR "Negros OR Bissexuais OR Bissexual OR Gay OR Gays OR Lésbica OR Lésbicas OR "Minorias Sexuais" OR LGBTQIA OR LGBTQIA+ OR LGBTQIAPN+ OR Homossexualidade OR "Homossexualidade Feminina" OR "Homossexualidade Masculina" OR "Identidade de Gênero")	311.597
#2	(Todos os campos:"Populações Vulneráveis" OR "Vulnerabilidade Social")	3.066
#3	(Todos os campos:"Desigualdades de Saúde")	307
#4	#2 AND #3	12
#5	(Todos os campos: Ensino OR Currículo OR "Educação Continuada" OR "Educação Profissionalizante" OR "Educação Interprofissional" OR "Capacitação Profissional" OR "Instituições Acadêmicas" OR "Pessoal de Saúde")	150.175
#6	#1 AND #4 AND #5	01

Networked Digital Library of Theses and Dissertations – NDLTD

SEARCH	QUERY	RECORDS RETRIEVE D
#1	("Black People" OR "Black Peoples" OR "Black Population" OR blacks OR "American Negro" OR black OR "LGBT Person" OR Gays OR Gay OR Lesbians OR Lesbian OR LGBTQIA+ people OR LGBTQIAPN+ OR "Gender Identity") AND ("Vulnerable Populations" OR "Social Vulnerability" OR Vulnerability OR Vulnerable) AND "Health Inequities" AND (Teaching OR Curriculum OR "Education, Continuing" OR "Education, Professional" OR "Interprofessional Education" OR "Professional Training" OR Schools OR "Health Personnel")	03

ProQuest Dissertations & Theses Global – PQDT (Clarivate)

SEARCH	QUERY	RECORDS RETRIEVE D
S1	title("Black People" OR "Black Peoples" OR "People, Black" OR "Black Person" OR "Black Persons" OR "Negroid Race" OR "Negroid Races" OR "Race, Negroid" OR "African Continental Ancestry Group") OR abstract("Black People" OR "Black Peoples" OR "People, Black" OR "Black Person" OR "Black Persons" OR "Negroid Race" OR "Negroid Races" OR "Race, Negroid" OR "African Continental Ancestry Group") OR title("Black Population" OR "Black man" OR "Black race" OR Negroid OR "Negroid race" OR Negroids) OR abstract("Black Population" OR "Black man" OR "Black race" OR Negroid OR "Negroid race" OR Negroids) OR title("Black or African American" OR "Black Americans" OR "American, Black" OR "Black American" OR Blacks OR Negroes OR Negro OR "African Americans" OR "African American" OR "American, African" OR "Afro-American" OR "Afro American" OR "Afro-Americans" OR "Afro Americans" OR "African-Americans" OR "African-American") OR abstract("Black or African American" OR "Black Americans" OR "American, Black" OR "Black American" OR Blacks OR Negroes OR Negro OR "African Americans" OR "African American" OR "American, African" OR "Afro-American" OR "Afro American" OR "Afro-Americans" OR "Afro Americans" OR "African-Americans" OR "African-American") OR title("American blacks" OR "American Negro" OR "black American" OR "black or African American") OR abstract("American blacks" OR "American Negro" OR "black American" OR "black or African American")	130.732
S2	(title("Sexual and Gender Minorities" OR "LGBT Person" OR "Persons, LGBT" OR "LGBTQ Person" OR "Person, LGBTQ" OR "Persons, LGBTQ" OR "Non-Heterosexual Persons" OR "Non Heterosexual Persons" OR "LBG Persons" OR "Sexual Minorities" OR "Minorities, Sexual" OR "Minority, Sexual" OR "Sexual Minority" OR "Non-Heterosexuals" OR "Non Heterosexuals" OR "Non-Heterosexual" OR "Sexual Dissidents" OR "Sexual Dissident" OR "GLBT Persons" OR "GLBT Person" OR Gays OR Gay OR "Men Who Have Sex With Men" OR Lesbians OR Lesbian OR "Women Who Have Sex With Women" OR Homosexuals OR Homosexual) OR abstract("Sexual and Gender Minorities" OR "LGBT Person" OR "Persons, LGBT" OR "LGBTQ Person" OR "Person, LGBTQ" OR "Persons, LGBTQ" OR "Non-Heterosexual Persons" OR "Non Heterosexual Persons" OR "LBG Persons" OR "Sexual Minorities" OR "Minorities, Sexual" OR "Minority, Sexual" OR "Sexual Minority" OR "Non-Heterosexuals" OR "Non Heterosexuals" OR "Non-Heterosexual" OR "Sexual Dissidents" OR "Sexual Dissident" OR "GLBT Persons" OR "GLBT Person" OR Gays OR Gay OR "Men Who Have Sex With Men" OR Lesbians OR Lesbian OR "Women Who Have Sex With Women" OR Homosexuals OR Homosexual) OR title("sexual and gender minority") OR abstract("sexual and gender minority") OR title("LGBTQIA+ people" OR pansexual OR questioning OR " transgender and intersex" OR transgender OR "GLBTI+" OR "GLBTQ+" OR "LGBTI+" OR "LGBTIQ+" OR "LGBTIQA+" OR "LGBTIQQ" OR "LGBTQ people" OR "LGBTQ+" OR "LGBTQ2" OR "LGBTQ2S" OR "LGBTQ2SIA+" OR "LGBTQA" OR "LGBTQAI" OR "LGBTQIA" OR "LGBTQIA+" OR "LGBTQIA2S+" OR "LGBTQQ" OR "LGBTQQIA" OR "LGBTTQ+" OR "LGTBQA" OR asexual OR "two-spirit" OR "2-spirit" OR "2 spirit") OR abstract("LGBTQIA+ people" OR pansexual OR questioning OR " transgender and intersex" OR transgender OR "GLBTI+" OR "GLBTQ+" OR "LGBTI+" OR "LGBTIQ+" OR "LGBTIQA+" OR "LGBTIQQ" OR "LGBTQ people" OR "LGBTQ+" OR "LGBTQ2" OR "LGBTQ2S" OR "LGBTQ2SIA+" OR "LGBTQA" OR "LGBTQAI" OR "LGBTQIA" OR	41.153

	"LGBTQIA+" OR "LGBTQIA2S+" OR "LGBTQQ" OR "LGBTQQIA" OR "LGBTTQ+" OR "LGTBQA" OR asexual OR "two-spirit" OR "2-spirit" OR "2 spirit") OR title(LGBTQIAPN+) OR abstract(LGBTQIAPN+) OR title(Homosexuality OR "Ego-Dystonic Homosexuality") OR abstract(Homosexuality OR "Ego-Dystonic Homosexuality")) OR (title("Homosexuality, Female" OR "Female Homosexuality" OR Lesbianism) OR abstract("Homosexuality, Female" OR "Female Homosexuality" OR Lesbianism) OR title("Homosexuality, Male" OR "Male Homosexuality") OR abstract("Homosexuality, Male" OR "Male Homosexuality") OR title("gender nonbinary" OR "gender non binary" OR "gender non conforming" OR "gender nonconforming" OR "non binary AFAB" OR "non binary gender" OR "non binary gender identity" OR "non binary individuals" OR "non binary people" OR "non conforming gender" OR "nonbinary AFAB" OR "nonbinary gender" OR "nonbinary individuals" OR "nonbinary people") OR abstract("gender nonbinary" OR "gender non binary" OR "gender non conforming" OR "gender nonconforming" OR "non binary AFAB" OR "non binary gender" OR "non binary gender identity" OR "non binary individuals" OR "non binary people" OR "non conforming gender" OR "nonbinary AFAB" OR "nonbinary gender" OR "nonbinary individuals" OR "nonbinary people") OR title("gender expansive youth" OR "gender-expansive people" OR "gender-fluid" OR "gender-queer" OR "gender-questioning" OR "genderexpansive" OR genderfluid OR genderqueer OR genderquestioning OR "TGNB people" OR "TNB individuals" OR "TNB people" OR "transgender and gender non-conforming" OR "transgender and gender nonconforming" OR "transgender and nonbinary") OR abstract("gender expansive youth" OR "gender-expansive people" OR "gender-fluid" OR "gender-queer" OR "gender-questioning" OR "genderexpansive" OR genderfluid OR genderqueer OR genderquestioning OR "TGNB people" OR "TNB individuals" OR "TNB people" OR "transgender and gender non-conforming" OR "transgender and gender nonconforming" OR "transgender and nonbinary") OR title("Gender Identity") OR abstract("Gender Identity"))	
S3	S1 OR s2	169.027
S4	title("Vulnerable Populations" OR "Vulnerable Population" OR "Disadvantaged Populations" OR "Disadvantaged Population" OR "Sensitive Populations" OR "Sensitive Population" OR "Sensitive Population Groups" OR "Sensitive Population Group" OR "Underserved Population" OR "Underserved Populations") OR abstract("Vulnerable Populations" OR "Vulnerable Population" OR "Disadvantaged Populations" OR "Disadvantaged Population" OR "Sensitive Populations" OR "Sensitive Population" OR "Sensitive Population Groups" OR "Sensitive Population Group" OR "Underserved Population" OR "Underserved Populations") OR title("vulnerable minorities" OR "vulnerable minority" OR "vulnerable minority population" OR "vulnerable people" OR "vulnerable person" OR "vulnerable persons") OR abstract("vulnerable minorities" OR "vulnerable minority" OR "vulnerable minority population" OR "vulnerable people" OR "vulnerable person" OR "vulnerable persons") OR title("Social Vulnerability" OR "Social Vulnerabilities") OR abstract("Social Vulnerability" OR "Social Vulnerabilities") OR title(Vulnerability OR Vulnerable) OR abstract(Vulnerability OR Vulnerable)	64.754
S5	title("Health Inequities" OR "Health Inequity" OR "Health Inequalities" OR "Health Inequality" OR "Health Disparities" OR "Health Disparity") OR abstract("Health Inequities" OR "Health Inequity" OR "Health Inequalities" OR "Health Inequality" OR "Health Disparities" OR "Health Disparity") OR title("disparity in health" OR "health status disparities" OR "health status disparity" OR "inequality in health" OR "inequity in health" OR "socioeconomic disparities in health") OR abstract("disparity in health" OR "health status disparities" OR "health status disparity" OR "inequality in health" OR "inequity in health" OR "socioeconomic disparities in health")	4.755
S6	(title(health) OR abstract(health)) AND (title(Inequities OR Inequity OR Inequalities OR Inequality OR Disparities OR Disparity) OR abstract(Inequities OR Inequity OR Inequalities OR Inequality OR Disparities OR Disparity))	14.915
**S7**	**S5 OR S6**	**14.915**
**S8**	**S4 AND S7**	**1.572**

S9	(title("Interprofessional Education" OR "Education, Interprofessional") OR abstract("Interprofessional Education" OR "Education, Interprofessional") OR title("inter-professional education" OR "cross training" OR multi-skilling OR multiskilling) OR abstract("inter-professional education" OR "cross training" OR multi-skilling OR multiskilling) OR title("Professional Training") OR abstract("Professional Training") OR title(Schools OR School OR "Secondary School" OR "Secondary Schools") OR abstract(Schools OR School OR "Secondary School" OR "Secondary Schools") OR title("Health Personnel" OR "Healthcare Workers" OR "Healthcare Worker" OR "Health Care Providers" OR "Health Care Provider" OR "Healthcare Providers" OR "Healthcare Provider" OR "Health Care Professionals" OR "Health Care Professional") OR abstract("Health Personnel" OR "Healthcare Workers" OR "Healthcare Worker" OR "Health Care Providers" OR "Health Care Provider" OR "Healthcare Providers" OR "Healthcare Provider" OR "Health Care Professionals" OR "Health Care Professional")) OR (title(Teaching OR "Training Techniques" OR "Training Technique" OR "Training Technics" OR "Training Technic" OR Pedagogy OR Pedagogies OR "Teaching Methods" OR "Teaching Method" OR "Academic Training" OR "Training Activities" OR "Training Activity" OR "Educational Techniques" OR "Educational Technique" OR "Educational Technics" OR "Educational Technic") OR abstract(Teaching OR "Training Techniques" OR "Training Technique" OR "Training Technics" OR "Training Technic" OR Pedagogy OR Pedagogies OR "Teaching Methods" OR "Teaching Method" OR "Academic Training" OR "Training Activities" OR "Training Activity" OR "Educational Techniques" OR "Educational Technique" OR "Educational Technics" OR "Educational Technic") OR title(Curriculum OR Curricula OR "Short-Term Courses" OR "Short Term Courses") OR abstract(Curriculum OR Curricula OR "Short-Term Courses" OR "Short Term Courses") OR title("competency-based education" OR "integrated curriculum") OR abstract("competency-based education" OR "integrated curriculum") OR abstract("Education, Continuing" OR "Continuous Learning" OR "Lifelong Learning" OR "Life-Long Learning" OR "Life Long Learning" "Continuing Education") OR title("Education, Continuing" OR "Continuous Learning" OR "Lifelong Learning" OR "Life-Long Learning" OR "Life Long Learning" "Continuing Education") OR title("Education, Professional" OR "Professional Education") OR abstract("Education, Professional" OR "Professional Education"))	606.102
**S10**	**S3 AND S8 AND S9**	**57**

Supplementary Matrial SII - Data Extraction Instrument

Supplementary Matrial SII A: For primary studies[1]

**Author(s)**	
**Year of publication**	
Study Design (Type)	
Objective(s)	
Context	Region/State
	Developed Location (University, Health Service, Community, etc.)

[1] workers and future health workers or institutions and initiatives, supplementary material II A and B, respectively, see participants.

Participants (Health Workers and Future Health Professionals)	Occupation/Field of Expertise
	Race/Ethnicity
	Sex/Gender Identity
	Age (Mean/Median)
	Educational Level
Concept	Topics Covered: HBP or Health of the LGBTQIA+ Population
	Methodological Approach
	Course, Subject, or Class (Curricular or Standalone Initiative)
	Objective of the Educational Approach
	Results achieved
	Limitations and Recommendations

Supplementary Matrial SII B: For theses and dissertations

**Author(s)**	
**Year of publication**	
Study Design (Type)	
Objective(s)	
Context	Region/State
	Setting (University, health service, community, etc.)
Participants (Institutions and Initiatives	Type: University, Research Centers, Health Services: health centers, hospitals, outpatient clinics.
	Sector: Public, private, philanthropic, or mixed institution
	Field: Health, Education, Research, Assistance, Management.
Concept	Topics Covered: HBP or Health of the LGBTQIA+ Population<br>HBP or Health of the LGBTI+ population
	Methodological Approach
	Course, Subject, or Class (Curricular or Standalone Initiative)
	Objective of the Educational Approach
	Results achieved
	Limitations and Recommendations

Supplementary material SII C: For systematic and scoping reviews

<table>
<tr><th colspan="2">Author(s)</th></tr>
<tr><th colspan="2">Year of publication</th></tr>
<tr><td colspan="2">Objective(s) of the Review</td></tr>
<tr><td colspan="2">"Eligibility Criteria for the Population (as defined by the authors for population selection.</td></tr>
<tr><td colspan="2">Eligibility Criteria for Study Selection (as defined by the authors)</td></tr>
<tr><td colspan="2">Eligibility Criteria for Publication (language, publication period/year, location)</td></tr>
<tr><td colspan="2">Number of studies included</td></tr>
<tr><td rowspan="4">Concept</td><td>Topics Covered: HBP or Health of the LGBTQIA+ Population
HBP or Health of the LGBTI+ population</td></tr>
<tr><td>Methodological Approach</td></tr>
<tr><td>Course, Subject, or Class (curricular or standalone initiative)</td></tr>
<tr><td>Objective of the Educational Approach</td></tr>
<tr><td colspan="2">Results achieved</td></tr>
<tr><td colspan="2">Limitations and Recommendations</td></tr>
</table>

Supplementay Material SIII: Thematic gap matrix by region and level of education.

Region / Education Level	Technical Training	Undergraduate Education	Postgraduate Education	Continuing Education / In-Service Training
North America				
South America				
Europe				

Africa

Asia

Oceania

Multiregional /
Global
